# PAI-1 derived from cancer-associated fibroblasts in esophageal squamous cell carcinoma promotes the invasion of cancer cells and the migration of macrophages

**DOI:** 10.1038/s41374-020-00512-2

**Published:** 2020-12-12

**Authors:** Hiroki Sakamoto, Yu-ichiro Koma, Nobuhide Higashino, Takayuki Kodama, Kohei Tanigawa, Masaki Shimizu, Masataka Fujikawa, Mari Nishio, Manabu Shigeoka, Yoshihiro Kakeji, Hiroshi Yokozaki

**Affiliations:** 1 Division of Pathology, Department of Pathology, Kobe University Graduate School of Medicine, 7-5-1 Kusunoki-cho, Chuo-ku, Kobe, 650-0017 Japan; 2 Division of Gastro-intestinal Surgery, Department of Surgery, Kobe University Graduate School of Medicine, 7-5-2 Kusunoki-cho, Chuo-ku, Kobe, 650-0017 Japan

**Keywords:** Cancer microenvironment, Oesophageal cancer, Oncogenesis

## Abstract

Cancer-associated fibroblasts (CAFs) contribute to the progression of various cancers. Previously, we reported the significance of CAFs in esophageal squamous cell carcinoma (ESCC); however, the functions of CAFs in the ESCC microenvironment remain unknown. To investigate CAFs’ function, we established an indirect coculture assay between human bone marrow-derived mesenchymal stem cells (MSCs) and ESCC cells. Cocultured MSCs expressed more fibroblast activation protein, one of the markers of CAFs, compared with monocultured MSCs. Therefore, we defined cocultured MSCs as CAF-like cells. To identify molecules associated with the ESCC progression in CAFs, we conducted a cDNA microarray analysis on monocultured MSCs and CAF-like cells to compare their gene expression profiles. We found that *SERPINE1*, which encodes plasminogen activator inhibitor-1 (PAI-1), was more abundant in CAF-like cells than in monocultured MSCs, and the PAI-1 derived from CAF-like cells induced the abilities of migration and invasion in both ESCC cells and macrophages by the Akt and Erk1/2 signaling pathways via the low-density lipoprotein receptor-related protein 1 (LRP1), which is a PAI-1 receptor. Based on immunohistochemistry assays of ESCC tissues, higher expression levels of PAI-1 and LRP1 were correlated with poor prognosis in ESCC patients. These results suggest that the PAI-1/LRP1 axis contributes to the progression of ESCC, making it a potential target for ESCC therapy.

## Abstract (web summary)

The authors show that plasminogen activator inhibitor-1 (PAI-1) derived from cancer-associated fibroblasts promotes the invasion of esophageal squamous cell carcinoma (ESCC) cells and the migration of macrophages *via* lipoprotein receptor-related protein 1 (LRP1). High expression of PAI-1 and/or LRP1 is associated with poor prognosis in patients with ESCC, and the PAI-1/LRP1 axis could be a target of anticancer therapy.

## Introduction

Globally, esophageal cancer is the seventh most common cancer and the sixth cause of cancer-related deaths. Esophageal cancer consists of two main histological types, including esophageal squamous cell carcinoma (ESCC) and esophageal adenocarcinoma. ESCC is the predominant histological type in east Asia, eastern and southern Africa, and southern Europe [1, 2]. Important risk factors for ESCC development are alcohol use, smoking, achalasia, race, and a high-starch diet, but the detailed mechanism remains unclear [3].

The tumor microenvironment (TME) can foster tumor progression. The TME includes various stromal cells, such as cancer-associated fibroblasts (CAFs), tumor-associated macrophages (TAMs), and other nonmalignant cells; and extracellular components (cytokines, growth factors, extracellular matrix, etc.) [4]. CAFs play key roles in tumor progression by mediating the activation of tumor proliferation, migration, and invasion, as well as the induction of angiogenesis, the stimulation of metastasis, and more [5, 6]. CAFs potentially originate from bone marrow-derived mesenchymal stem cells (MSCs), resident fibroblasts, endothelial cells, adipocytes, and epithelial cells [7]. The heterogeneous origin of CAFs accounts for their broad range of characteristics and their various molecular markers [8]. Alpha-smooth muscle actin (αSMA) and fibroblast activation protein (FAP) are representative cellular markers of CAFs, and their elevated expressions are associated with poorer prognosis in several cancers [9, 10]. Moreover, recent studies indicate that CAFs indirectly contribute to tumor progression by interacting with other stromal components of the TME such as TAMs [11, 12].

In a previous study, we characterized the mechanisms of tumor-promoting ability and immunosuppressive phenotype of CAFs in ESCC [13]. We observed that higher expression levels of αSMA and FAP in the tumor stroma were significantly associated with increasing depth of tumor invasion and lymph node metastasis, advanced pathological stage, and poorer prognosis in patients with ESCC. In addition, we performed a coculture assay between MSCs and ESCC cells and found an increase of FAP expression in the cocultured MSCs. These FAP-positive MSCs, which we defined as CAF-like cells, promoted the migrative abilities of both ESCC cells and macrophages by secreting C-C motif chemokine 2 (CCL2) and interleukin-6 (IL6) and induced the M2 polarization of macrophages. However, the interactions among CAFs, M2-polarized macrophages (so-called TAMs), and ESCC cells have not been clarified in detail.

To explore the mechanisms by which CAFs enhance ESCC progression, we established CAF-like cells by coculturing MSCs with ESCC cells in vitro. We then compared the gene expression profiles between monocultured MSCs and CAF-like cells by cDNA microarray analysis and characterized the effects of CAF-like cells to ESCC cells and macrophages. Moreover, we identified possible key players underlying the tumor-promoting effects of CAF-like cells in the ESCC microenvironment that may be used as potential targets for ESCC therapy.

## Materials and methods

### Cell lines and cell cultures

Three human ESCC cell lines, TE-series ESCC cells (TE-8, TE-9, and TE-15 cells), were purchased from RIKEN BioResource Center (Tsukuba, Japan) and were cultured in RPMI-1640 (FUJIFILM Wako Pure Chemical, Osaka, Japan) containing 10% FBS (Sigma-Aldrich, St. Louis, MO, USA) and 1% antibiotic-antimycotic (Invitrogen, Carlsbad, CA, USA) [14]. The human MSCs (ATCC® PCS-500-012^TM^) were purchased from the American Type Culture Collection (Manassas, VA, USA) and were cultured in DMEM with low-glucose (FUJIFILM Wako Pure Chemical) containing 10% FBS and 1% antibiotic-antimycotic [13].

### Preparation of CAF-like cells and macrophages

The method for the establishment of CAF-like cells was described in a previous report [13]. In brief, MSCs (5 × 10^4^ cells) were seeded in the lower chamber of a 6-well plate and cocultured with ESCC cells (1.5 × 10^5^ cells) in the upper chamber (0.4-μm pore size insert; BD Falcon, Lincoln Park, NY, USA) for 7 days. The media and the upper chamber with ESCC cells were changed 4 days after coculturing. As a control, MSCs were seeded without ESCC cells.

We induced macrophages from CD14^+^ peripheral blood monocytes (PBMos) as previously described [15, 16]. In brief, PBMos were purified from peripheral blood mononuclear cells by positive selection using an autoMACS® Pro Separator (Miltenyi Biotec, Bergisch Gladbach, Germany). Then, PBMos (5 × 10^5^ cells) in the 6-well plate were cultured with macrophage-colony stimulating factor (M-CSF; 25 ng/ml; R&D Systems, Minneapolis, MN, USA) for 6 days to induce macrophages.

### cDNA microarray analysis

The extraction of total RNA from MSCs or CAF-like cells was performed by the RNeasy Mini Kit (Qiagen, Hilden, Germany). We conducted cDNA microarray analysis using the 3D-Gene® Human Oligo chip 25k (Toray Industries, Tokyo, Japan). Microarray slides were scanned using the 3D-Gene® Scanner (Toray Industries) and processed using the 3D-Gene® Extraction software (Toray Industries). We have deposited these data in the Gene Expression Omnibus database (GSE143138).

### Reverse Transcription-PCR (RT-PCR) and quantitative RT-PCR (qRT-PCR)

The extraction of total RNA from each cell was performed by the RNeasy Mini Kit (Qiagen). We performed RT-PCR and qRT-PCR as previously reported [13]. RT-PCR products of *LRP1* and *GAPDH* (control) were separated by electrophoresis in agarose gel (2%). We conducted qRT-PCR amplifications of *FAP*, *SERPINE1, IL6, CCL2, CXCL12, CD163, MSR1, CD274*, and *ACTB* (control) on the StepOne^TM^ Real-Time PCR System (Thermo Fisher Scientific, Waltham, MA, USA) using Taqman® Gene Expression Master Mix (Thermo Fisher Scientific). Primers and probes are listed in Table S1.

### Western blotting

Methods to extract cellular proteins and western blotting are described elsewhere [13]. In brief, cells were lysed on ice with a RIPA Lysis and Extraction Buffer (Thermo Fisher Scientific) containing 1% protease inhibitor and 1% phosphatase inhibitor cocktail (Sigma-Aldrich). The resulting lysates were separated on 5–20% sodium dodecyl sulfate polyacrylamide gels and transferred to a membrane with an iBlot® Gel Transfer Stack (Invitrogen). The membrane was blocked with 5% skim milk and then incubated with primary and secondary antibodies. The protein bands were detected with ImmunoStar Reagents (FUJIFILM Wako Pure Chemical). The lists of primary and secondary antibodies are presented in Table S2. Densitometric analysis of the bands obtained in western blotting was performed using the Wand (tracing) tool in the ImageJ software (version 1.52a; National Institutes of Health, Bethesda, MD, USA), and the levels of phospho-proteins were normalized to total protein levels.

### ELISA

The culture media of monocultured MSCs, CAF-like cells, and ESCC cells were exchanged to serum-free DMEM. After 24 h, the supernatants were collected and analyzed by Human Serpin E1/PAI-1 Quantikine® ELISA Kit (R&D Systems) in accordance with the manufacturer’s instructions. The optical densities of each well were read at 450 and 570 nm using a microplate reader (Infinite® 200 PRO; Tecan, Männedorf, Switzerland). The PAI-1 concentration in each well was calculated from the absorbance values using a standard curve.

### Transwell migration assay and transwell invasion assay

We performed the transwell migration assay and transwell invasion assay using an 8.0-µm pore size insert (BD Falcon) and a BioCoat^TM^ Matrigel® Invasion Chamber (Corning, Tewksbury, MA, USA), respectively. To investigate the effects of the coculture, CAF-like cells (5 × 10^4^ cells) in serum-free media were seeded in the lower chambers. The effect of recombinant human PAI-1 (rhPAI-1; R&D Systems) was investigated by addition to the lower chambers. ESCC cells (1 × 10^5^ cells for transwell migration assay; 3 × 10^5^ cells for transwell invasion assay) or macrophages (1 × 10^5^ cells for transwell migration assay; 3 × 10^5^ cells for transwell invasion assay) in the serum-free media were seeded in the upper inserts. After 24 h or 48 h, the cells that migrated through the membranes were stained using Diff-Quik® (Sysmex, Kobe, Japan) and counted. In some experiments, ESCC cells or macrophages were treated with PI3K inhibitor LY294002 (Cell Signaling Technology, Beverly, MA, USA) or MEK1/2 inhibitor PD98059 (Cell Signaling Technology); CAF-like cells were treated with neutralizing antibody against human PAI-1 (AF1786; R&D Systems) or normal goat IgG (AB-108-C; R&D Systems) as the negative control.

### Wound healing assay

ESCC cells (TE-8 and TE-9 cells; 2 × 10^5^ cells) in RPMI-1640 (FUJIFILM Wako Pure Chemical) containing 10% FBS (Sigma-Aldrich) were seeded in a 24-well plate. After 24 h, the confluent cell monolayer was wounded by mechanical scratching and exchanged into serum-free media with or without rhPAI-1 at 10 ng/mL (R&D Systems). After another 24 h, we observed cells migrating to the wound area and calculated the percent wound coverage using the Polygon selection tool in the ImageJ software (National Institutes of Health). We excluded TE-15 cells from the wound healing assay based on their status as nest-forming and nonconfluent cells.

### Knockdown of LRP1 by siRNA

ESCC cells or macrophages were transfected with 20 nM siRNA against human *LRP1* (siLRP1, sc-40101; Santa Cruz Biotechnology, Dallas, TX, USA) or 20 nM negative control siRNA (siNC, S1C-001; Sigma-Aldrich) for 48 h using Lipofectamine® RNAiMAX (Invitrogen) in accordance with the manufacturer’s instructions. After transfection, the cells were used for in vitro experiments.

### Tissue samples

A total of 69 surgically resected cases of human ESCC tissues at Kobe University Hospital (Kobe, Japan) from 2005 to 2010 were included in this study, as previously described [13, 17]. All patients had not received adjuvant chemotherapy or radiotherapy. Informed consent for the use of tissue samples and clinical data were obtained from all patients, and this study was approved by the Kobe University Institutional Review Board. Patient characteristics are presented in Table S3.

### Immunohistochemistry

Immunohistochemistry was performed on 4-µm tissue sections of paraffin-embedded specimens using EnVision Dual Link System-HRP (Agilent Technologies, Santa Clara, CA, USA) [13]. The list of primary antibodies is presented in Table S2. We used human placental tissue, which expressed both PAI-1 and LRP1 in villi, as a positive control and examined the condition of immunohistochemistry for these proteins (Fig. S1A). No immunoreactivity for normal rabbit IgG (sc-20271; Santa Cruz Biotechnology) was observed under the conditions used for the anti-PAI-1 and anti-LRP1 antibodies in human placental tissue (Fig. S1A) and human ESCC tissue (Fig. S1B, C). Qualitative scores for evaluating immunohistochemical staining intensities of PAI-1 in cancer stroma compared to those of vascular endothelial cells as positive control were as follows: negative to weak (low) and equal to strong (high). For LRP1 in cancer nests and cancer stroma were as follows: 0 (negative), 1 (weak), 2 (intermediate), and 3 (strong). Scores of 2 or 3 for LRP1 immunoreactivity were considered high. Two pathologists (YIK and HY) and one surgeon (HS), blinded to the patients’ clinical data, performed immunoreactivity scoring. We evaluated the proportion of immunohistochemical αSMA- and FAP-positive areas of the stroma around the invasive front per 40× field (4× objective and 10× ocular) as low (≤30%) and high (>30%) [13]. The method for the evaluation of CD163 and CD204 labeling of macrophages was described in a previous report [17]. The mean number of macrophages per 200× field (20× objective and 10× ocular) was calculated.

### Immunofluorescence

Double immunofluorescence was performed using anti-LRP1 and anti-CD204 antibodies in formalin-fixed paraffin-embedded human ESCC tissue. The nuclei were stained with DAPI (DOJINDO LABORATORIES, Kumamoto, Japan). The lists of primary and secondary antibodies are presented in Table S2. All images were taken with a Zeiss LSM 700 laser-scanning microscope and analyzed using the LSM software ZEN 2009 (Carl Zeiss, Oberkochen, Germany).

### Statistical analysis

Relationships between the immunohistochemical results and clinicopathological factors were analyzed by *χ*^2^-tests. Statistical significance in the in vitro assays were determined by two-tailed Student’s *t* test. A *p* < 0.05 was considered statistically significant. Overall, disease-free and cause-specific survival (CSS) curves were estimated by the Kaplan–Meier method and compared using the log-rank test. Statistical analyses were carried out by SPSS Statistics version 22 (IBM, Chicago, IL, USA).

## Results

### Expression of PAI-1 by MSCs and CAF-like cells

**Fig. 1:**
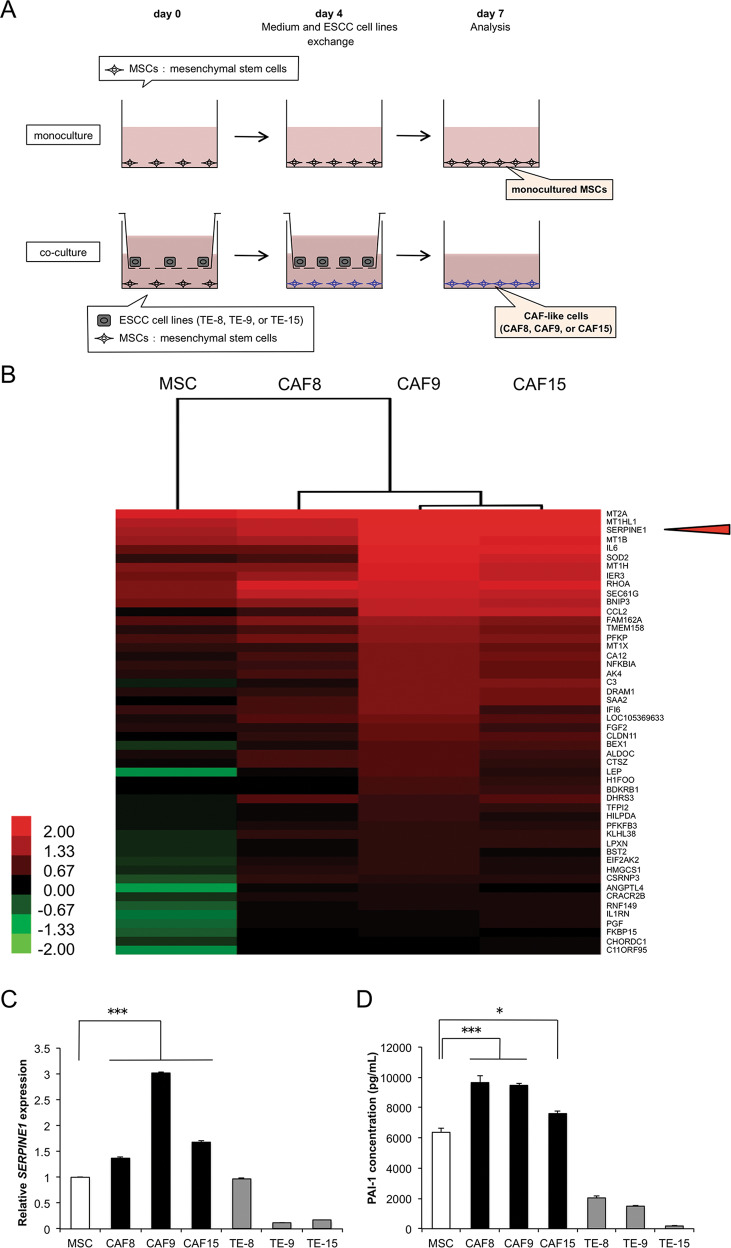
PAI-1 expression and secretion were induced in CAF-like cells. **a** Mesenchymal stem cells (MSCs) were cocultured with esophageal squamous cell carcinoma (ESCC) cell lines TE-8, TE-9, or TE-15 cells (herein defined as CAF-like cells, CAF8, CAF9, or CAF15, respectively) for 7 days. **b** Gene expression heatmap of monocultured MSCs and three CAF-like cells sorted in the descending order of up-regulated gene expression levels in CAF9 cells. Phylogenetic tree on top of the heatmap compares gene expression patterns of the different cell types. **c** Levels of *SERPINE1* mRNA in MSC, CAF-like cells, and ESCC cells were determined by quantitative RT-PCR and normalized relative to *ACTB* (*β-actin*). **d** Concentrations of PAI-1 in the supernatants of MSCs, CAF-like cells, and ESCC cells measured by ELISA. For (**c**) and (**d**), data represent the mean ± SEM of triplicate wells for three independent experiments (*p < 0.05, ***p < 0.001).

The functions of CAFs in the ESCC microenvironment were assessed by an indirect coculture assay (Fig. 1a). The mRNA and protein expression levels of FAP were induced in cocultured MSCs with three TE-series ESCC cells, TE-8, TE-9, and TE15 cells (Fig. S2A, B). Hereafter, we define cocultured MSCs with TE-8, TE-9, and TE-15 cells as CAF-like cells, CAF8, CAF9, and CAF15 cells, respectively (Fig. 1a). We compared gene expression profiles of monocultured MSCs and CAF-like cells by cDNA microarray analysis. We identified 110 genes that were up-regulated (ratio >2.0) and 128 genes that were down-regulated (ratio <0.5) in CAF9 (Table S4). The heatmap shows the top 50 genes with high expression among the up-regulated genes in CAF9 (Fig. 1b). The phylogenetic analysis revealed that the gene expression patterns in CAF9 and CAF15 were the most similar among the different cell groups. The monocultured MSCs had an expression pattern different from the CAF-like cells.

In this study, we focused on *serine protease inhibitor E1* (*SERPINE1*), one of the up-regulated, and highly expressed genes in CAF-like cells (Fig. 1b, Table 1). We confirmed the high expression levels of *SERPINE1* mRNA as well as those of *IL6*, *CCL2*, and *CXCL12* mRNA in CAF-like cells by qRT-PCR (Figs. 1c, S2C). The CAF-like cells secreted significantly higher concentrations of PAI-1 (which is encoded by *SERPINE1*) than monocultured MSCs (Fig. 1d). The results of the ELISA verified the secretion of PAI-1 from ESCC cells, especially from TE-8 and TE-9 cells (Fig. 1d).

**Table 1:** Up-regulated genes coding secretory proteins in CAF-like cells compared to monocultured MSCs.

Gene accession	Gene symbol	Gene description	Fold change		
			(CAF-like cells/monocultured MSCs)		
			CAF8	CAF9	CAF15
NM_000602.4	*SERPINE1*	*serpin peptidase inhibitor, clade E (nexin, plasminogen activator inhibitor type 1), member 1*	1.43	4.36	3.62
XM_011515390.1	*IL6*	*interleukin 6*	1.09	11.81	9.85
NM_002982.3	*CCL2*	*C-C motif chemokine ligand 2*	2.27	23.87	22.54

### Neutralizing antibody against PAI-1 suppresses CAF-like cells-induced migration of ESCC cells

**Fig. 2:**
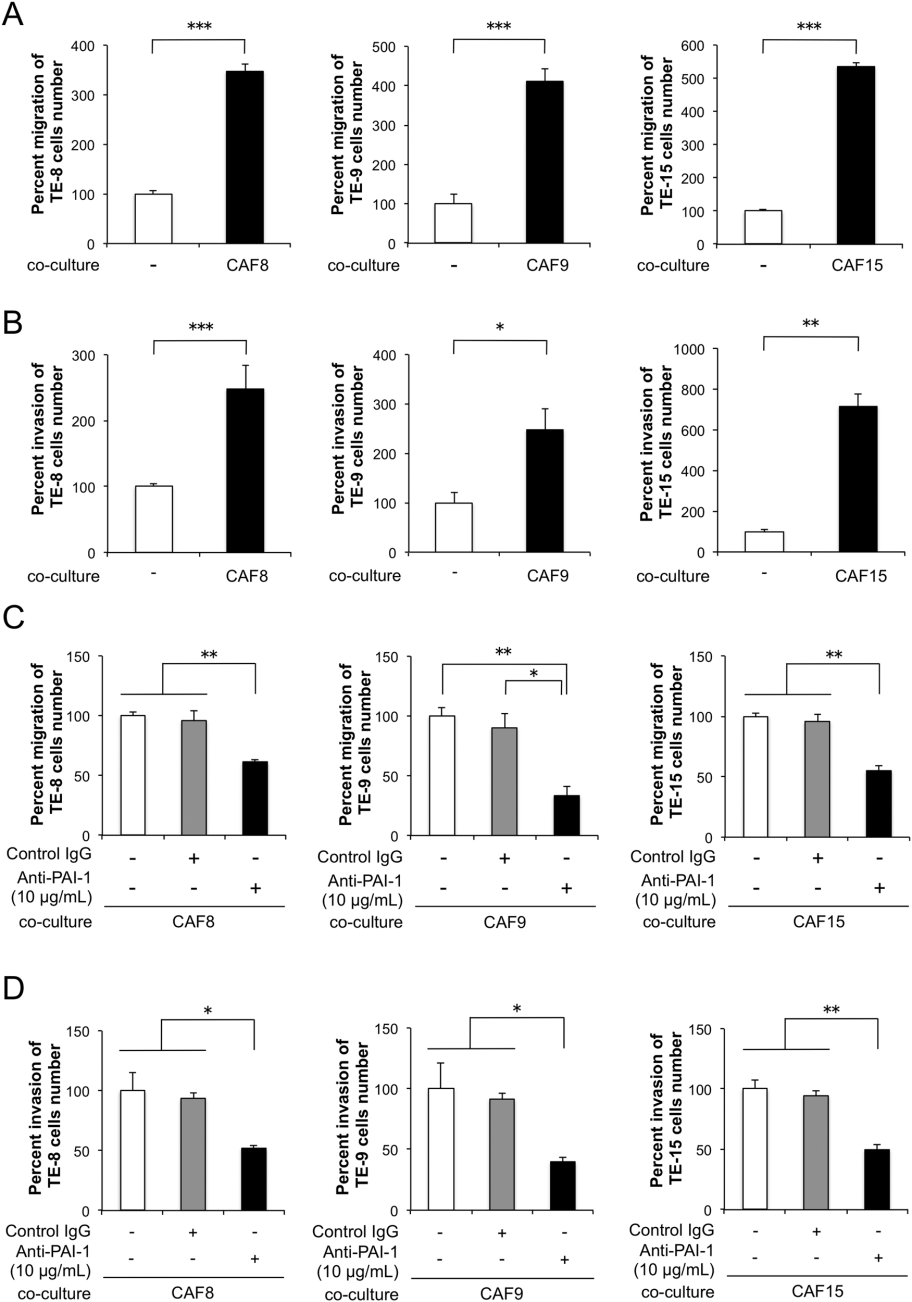
PAI-1 secreted by CAF-like cells promotes the migration and invasion of esophageal squamous cell carcinoma (ESCC) cells. **a** Transwell assay demonstrating the effects of CAF-like cells coculturing on the migration of TE-8, TE-9, and TE-15 cells. Migrating cells were counted in four randomly chosen fields. **b** Transwell assay demonstrating the effects of CAF-like cells coculturing on the invasion of TE-8, TE-9, and TE-15 cells. Invading cells were counted in four randomly chosen fields. **c** Transwell assay of the effects of PAI-1 neutralizing antibodies on the migration of TE-8, TE-9, and TE-15 cells cocultured with CAF-like cells. Normal goat IgG was the negative control for the PAI-1 antibody. **d** Transwell assay of the effects of PAI-1 neutralizing antibodies on the invasion of TE-8, TE-9, and TE-15 cells cocultured with CAF-like cells. Normal goat IgG was the negative control for the PAI-1 antibody. For (**a**–**d**), data represent the mean ± SEM of triplicate wells for three independent experiments (*p < 0.05, **p < 0.01, ***p < 0.001).

Coculture with CAF-like cells, CAF8, CAF9, and CAF15 cells, significantly induced the migration and invasion abilities of three TE-series ESCC cells, TE-8, TE-9, and TE-15 cells, respectively (Fig. 2a, b). The addition of a neutralizing antibody against PAI-1 significantly suppressed the migration and invasion abilities of ESCC cells cocultured with CAF-like cells (Fig. 2c, d).

### PAI-1 induces the migration and invasion of ESCC cells by activating Akt and Erk1/2 signaling pathways

**Fig. 3:**
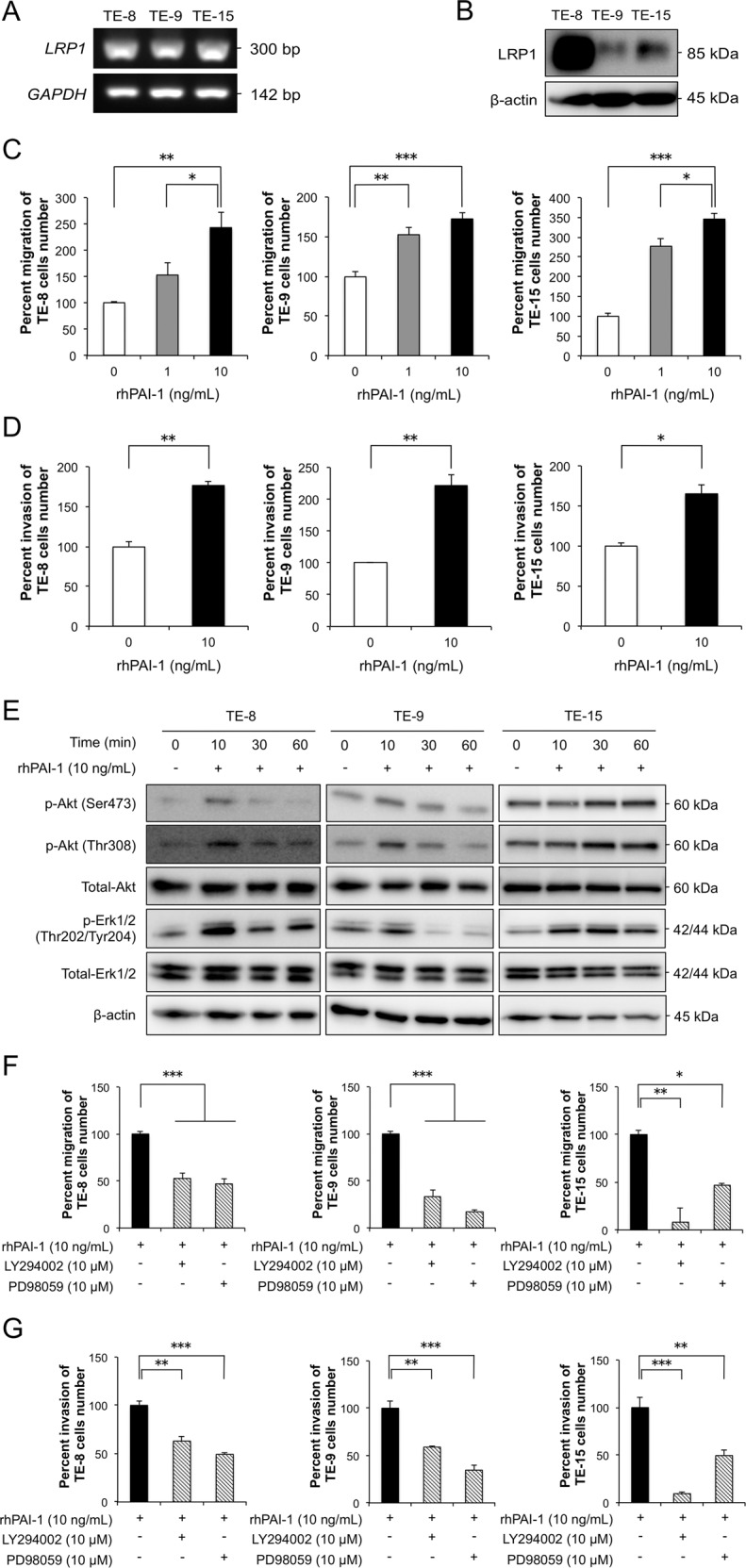
PAI-1 promotes the migration and invasion of esophageal squamous cell carcinoma (ESCC) cells by activating Akt and Erk1/2 signaling pathways. **a** Levels of *LRP1* mRNA in ESCC cells by RT-PCR. *GAPDH* used as internal control. **b** Expression of LRP1 in ESCC cells by western blotting. β-actin used as the loading control. **c** Transwell assay of the effects of recombinant human (rh) PAI-1 on the migration of TE-8, TE-9, and TE-15 cells. Migrating cells were counted in four randomly chosen fields. **d** Transwell assay of the effects of rhPAI-1 on the invasion of TE-8, TE-9, and TE-15 cells. Invading cells were counted in four randomly chosen fields. **e** Representative western blots of phosphorylated and total Akt and Erk1/2 in ESCC cells. TE-8, TE-9, and TE-15 cells in serum-free conditions were treated with 10 ng/mL rhPAI-1 for 0, 10, 30, and 60 min. **f** Transwell migration assay of TE-8, TE-9, and TE-15 cells with or without 10 ng/mL rhPAI-1 combined with an inhibitor against PI3K (LY294002, 10 μM) or MEK1/2 (PD98059, 10 μM). Migrating cells were counted in four randomly chosen fields. **g** Invasion assay of TE-8, TE-9, and TE-15 cells with or without 10 ng/mL rhPAI-1 combined with an inhibitor against PI3K (LY294002, 10 μM) or MEK1/2 (PD98059, 10 μM). Invading cells were counted in four randomly chosen fields. For (**c**), (**d**), (**f**), and (**g**), data represent the mean ± SEM of triplicate wells for three independent experiments (*p < 0.05, **p < 0.01, ***p < 0.001).

We confirmed the expression of LRP1, a PAI-1 receptor, in three TE-series ESCC cells using RT-PCR and western blotting (Fig. 3a, b). To investigate the effects of PAI-1 on the phenotypes and the signaling pathways of ESCC cells, we applied rhPAI-1 to TE-8, TE-9, and TE-15 cells. RhPAI-1 significantly promoted the migration and invasion abilities of ESCC cells (Fig. 3c, d). In addition, we confirmed that rhPAI-1 promoted the migration of TE-8 and TE-9 cells by wound healing assay (Fig. S3). RhPAI-1 increased p-Akt (Ser473/Thr308) and p-Erk1/2 levels after 10 or 30 min of treatment (Figs. 3e, S4). The PI3K inhibitor LY294002 and MEK1/2 inhibitor PD98059 suppressed the induction of both migration and invasion in rhPAI-1-treated ESCC cells (Fig. 3f, g).

### PAI-1 promotes the migration and invasion of ESCC cells and activates signaling pathways *via* LRP1

**Fig. 4:**
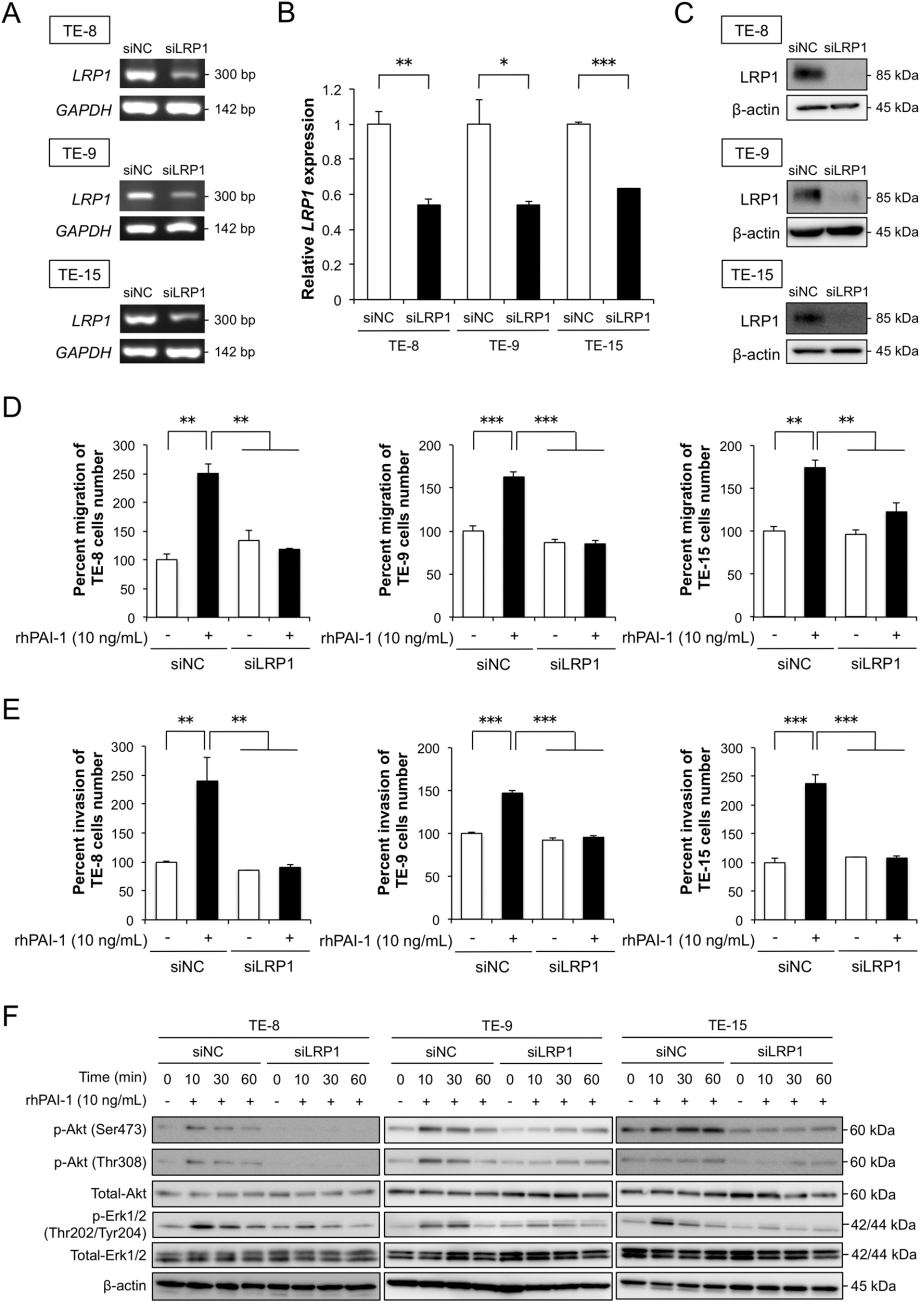
Effects of LRP1 knockdown on the migration, invasion, and signaling of esophageal squamous cell carcinoma (ESCC) cells. **a, b** ESCC cells were transfected with 20 nM siRNA targeting *LRP1* (siLRP1). Negative control siRNA (siNC) was transfected into ESCC cells as negative control. *LRP1* knockdown was confirmed by (**a**) RT-PCR with *GAPDH* as the internal control, and by (**b**) quantitative RT-PCR with *LRP1* normalized to levels in respective samples and *ACTB* (*β-actin*) as the internal control. **c** Representative western blots of LRP1 knockdown in ESCC cells. β-actin used as the loading control. **d** Transwell assay of the effects of LRP1 knockdown on the migration of TE-8, TE-9, and TE-15 cells with or without 10 ng/mL rhPAI-1. Migrating cells were counted in four randomly chosen fields. **e** Transwell assay of the effects of LRP1 knockdown on the invasion of TE-8, TE-9, and TE-15 cells with or without 10 ng/mL rhPAI-1. Invading cells were counted in four randomly chosen fields. **f** Representative western blots of the total protein of *LRP1*-silenced ESCC cells using specific antibodies against Akt, p-Akt (Ser473), p-Akt (Thr308), Erk1/2, p-Erk1/2 (Thr202/Tyr204), and β-actin compared to the control. For (**b**), (**d**), and (**e**), data represent the mean ± SEM of triplicate wells for three independent experiments (*p < 0.05, **p < 0.01, ***p < 0.001).

To clarify whether PAI-1 affects the phenotype and signaling of ESCC cells via LRP1, we suppressed *LRP1* mRNA in three TE-series ESCC cells by RNA interference. The silencing of the LRP1 was confirmed using RT-PCR, qRT-PCR, and western blotting (Fig. 4a, b, c). The knockdown of LRP1 in ESCC cells significantly suppressed the rhPAI-1-induced migration and invasion (Fig. 4d, e). The p-Akt and p-Erk1/2 levels decreased following LRP1 knockdown in ESCC cells (Figs. 4f and S5).

### PAI-1 induces the migration and invasion of macrophages by activating Akt and Erk1/2 signaling pathways via LRP1

**Fig. 5:**
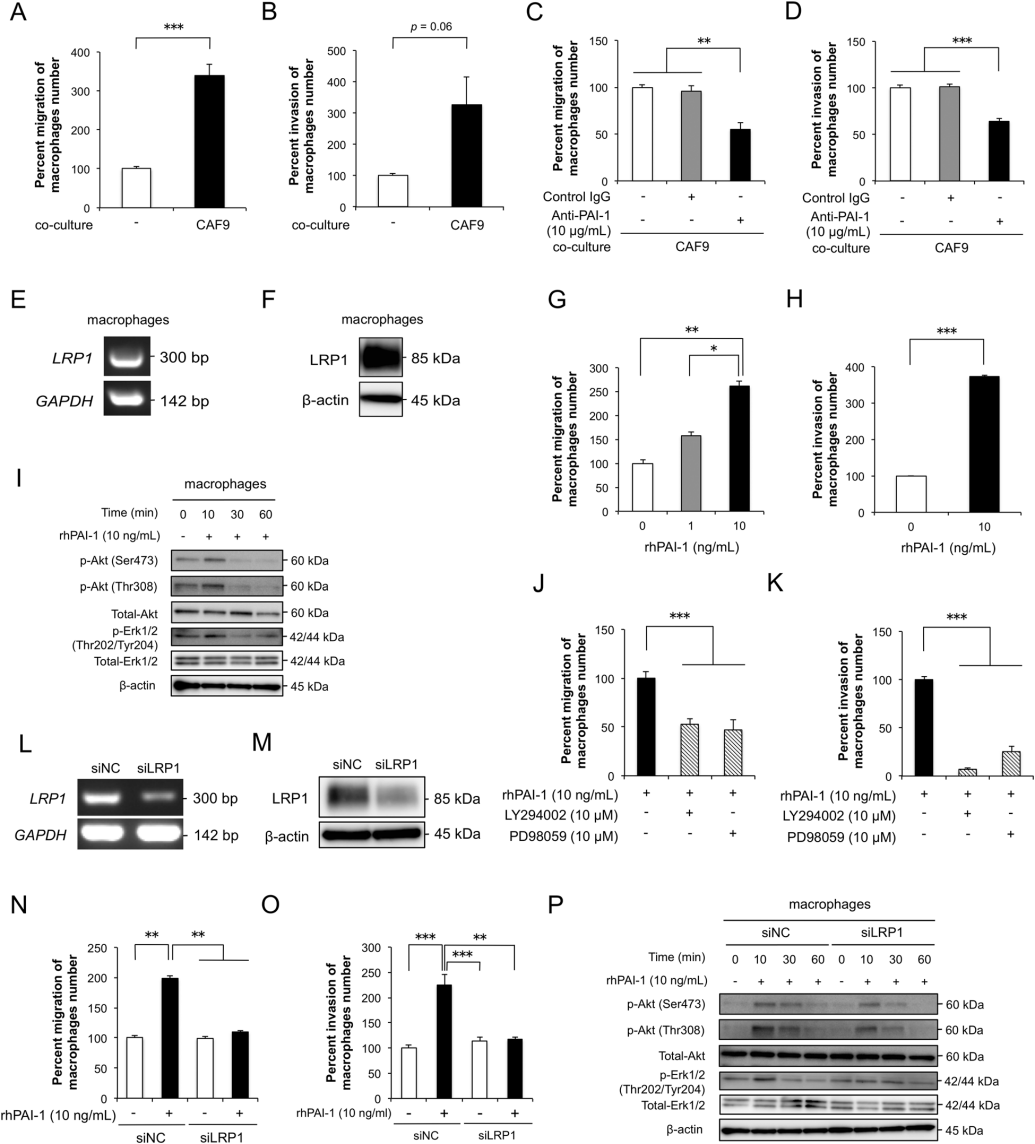
PAI-1 induces the migration and invasion of macrophages by activating Akt and Erk1/2 signaling pathways *via* LRP1. **a** Transwell assay of the effect of CAF9 cell coculturing on the migration of macrophages. **b** Transwell assay of the effect of CAF9 cell coculturing on the invasion of macrophages. **c** Transwell assay of the effect of a PAI-1 neutralizing antibody on the migration of macrophages cocultured with CAF9 cells. Normal goat IgG was used as negative control for the PAI-1 antibody. **d** Transwell assay of the effect of PAI-1 neutralizing antibody on the invasion of macrophages cocultured with CAF9. Normal goat IgG was used as negative control for the PAI-1 antibody. **e** The levels of *LRP1* mRNA in macrophages by RT-PCR using *GAPDH* as the control. **f** The expression level of LRP1 protein in macrophages by western blotting. Anti-LRP1 and β-actin antibodies were used. **g** Transwell assay of the effect of rhPAI-1 on the migration of macrophages. **h** Transwell assay of the effect of rhPAI-1 on the invasion of macrophages. **i** Representative western blots of the effect of rhPAI-1 on the phosphorylation levels of Akt and Erk1/2 in macrophages. Macrophages in serum-free conditions were treated with 10 ng/mL rhPAI-1 for 0, 10, 30, and 60 min. Western blotting was conducted with total protein extracted from macrophages using specific antibodies against Akt, p-Akt (Ser473), p-Akt (Thr308), Erk1/2, p-Erk1/2 (Thr202/ Tyr204), and β-actin. **j** Transwell migration assay of macrophages with or without 10 ng/mL rhPAI-1 combined with a PI3K inhibitor (LY294002, 10 μM) or a MEK1/2 inhibitor (PD98059, 10 μM). **k** Transwell invasion assay of macrophages with or without 10 ng/mL rhPAI-1 combined with a PI3K inhibitor (LY294002, 10 μM) or a MEK1/2 inhibitor (PD98059, 10 μM). **l** Macrophages were transfected with 20 nM siRNA targeting *LRP1* (siLRP1). siNC was transfected into macrophages as a negative control. *LRP1* knockdown was confirmed by RT-PCR using *GAPDH* as control. **m** Effective knockdown of LRP1 was confirmed by western blotting using antibodies against LRP1 and β-actin. **n** Transwell assay of the effects of LRP1 knockdown on the migration of macrophages with or without 10 ng/mL rhPAI-1. **o** Transwell assay of the effects of LRP1 knockdown on the invasion of macrophages with or without 10 ng/mL rhPAI-1. **p** Western blotting was performed on the total protein of *LRP1*-silenced macrophages using specific antibodies against Akt, p-Akt (Ser473), p-Akt (Thr308), Erk1/2, p-Erk1/2 (Thr202/ Tyr204), and β-actin compared to the control. For (**a–d**), (**g**), (**h**), (**j**), (**k**), (**n**), and (**o**), migrating and invading cells were counted in four randomly chosen fields, and data represent the mean ± SEM of triplicate wells for three independent experiments (*p < 0.05, **p < 0.01, ***p < 0.001).

We confirmed the effects of PAI-1 on macrophages by the same methods as above. Coculture with CAF-like cells significantly induced the migration and invasion of macrophages (Fig. 5a, b). Neutralizing antibodies against PAI-1 significantly suppressed the CAF-like cell-induced migration and invasion of macrophages (Fig. 5c, d). We confirmed the expression of LRP1 in macrophages using RT-PCR and western blotting (Fig. 5e, f). We found that rhPAI-1 significantly promoted the migration and invasion abilities of macrophages (Fig. 5g, H). We observed that rhPAI-1 increased p-Akt and p-Erk1/2 levels in macrophages after 10 min of treatment (Figs. 5i, S6A). However, rhPAI-1 did not affect the M2 polarization of macrophages (Fig. S7). LY294002 and PD98059 suppressed the abilities of migration and invasion in macrophages induced by rhPAI-1 (Fig. 5j, k). We next suppressed *LRP1* mRNA in macrophages by RNA interference. LRP1 silencing was confirmed by RT-PCR and western blotting (Fig. 5l, m), and knockdown of LRP1 in macrophages suppressed the rhPAI-1-induced migration and invasion (Fig. 5n, o). The levels of PAI-1-induced p-Akt and p-Erk1/2 were also reduced by the knockdown of LRP1 in macrophages (Fig. 5p, S6B).

### PAI-1 and LRP1 expression levels correlate with clinicopathological factors and prognosis of patients with ESCC

**Fig. 6:**
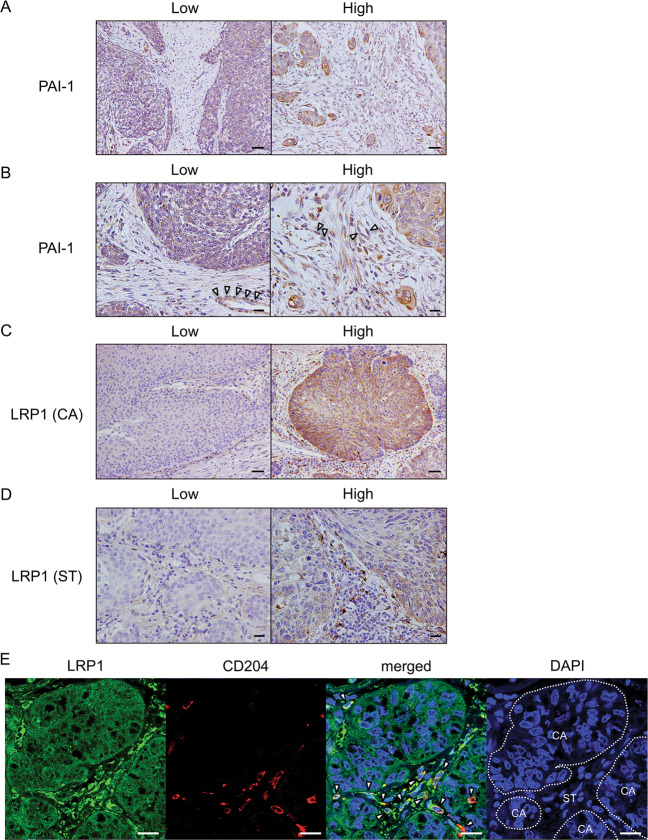
The expression of PAI-1 and LRP1 in human ESCC tissues. **a** Immunohistochemical staining for PAI-1 in human ESCC tissues. Representative images are shown: PAI-1 low-intensity in the stroma (left) and PAI-1 high-intensity in the stroma (right). Scale bars = 100 μm. **b** High-power fields of immunohistochemical staining for PAI-1 in same cases as Fig. 6a. Scale bars = 20 μm. Arrow heads: vascular endothelial cell. **c** Immunohistochemical staining for LRP1 in cancer nest of human ESCC tissues. Representative images are shown: LRP1 low-intensity in cancer nest (left) and LRP1 high-intensity in cancer nest (right). Scale bars = 100 μm. **d** Immunohistochemical staining for LRP1 in the stroma of human ESCC tissues. Representative images are shown: LRP1 low-intensity in the stroma (left) and LRP1 high-intensity in the stroma (right). Scale bars = 20 μm. **e** Stromal cells, including CD204^+^ TAMs, expressed LRP1 in human ESCC tissue. Double immunofluorescence was performed using anti-LRP1 (green) and anti-CD204 (red) antibodies in formalin-fixed paraffin-embedded section of human ESCC tissue. LRP1 was expressed in both cancer cells and stromal cells, and some of the LRP1-positive stromal cells were CD204^+^ macrophages (arrow heads). Nuclei were stained with DAPI (blue). Scale bar = 20 μm. CA cancer nest. ST stroma.

To clarify whether expression levels of PAI-1 and LRP1 were significantly associated with clinicopathological factors of patients with ESCC, we performed immunohistochemistry analyses of PAI-1 and LRP1 in 69 human ESCC tissues. We divided the ESCC tissues into high and low expression groups based on the immunoreactivity of PAI-1 in stromal cells and LRP1 in either cancer cells [LRP1 (CA)] or stromal cells [LRP1 (ST)] (Fig. 6a, b, c, d) and confirmed that stromal cells, including CD204^+^ TAMs, expressed LRP1 in human ESCC tissue by double immunofluorescence (Fig. 6e). We found that a high expression level of PAI-1 in the tumor stroma was closely correlated with depth of tumor invasion (*p* < 0.001), high expression levels of αSMA (*p* < 0.001) and FAP (*p* < 0.001), and high numbers of infiltrating CD204^+^ macrophages (*p* = 0.002) (Table 2). A high expression level of LRP1 (CA) was significantly correlated with a high expression level of FAP (*p* = 0.031) and high numbers of infiltrating CD204^+^ macrophages (*p* = 0.036) (Table 2). A high expression level of LRP1 (ST) was significantly correlated with sex (*p* = 0.031), depth of tumor invasion (*p* = 0.024), high expression levels of αSMA (*p* < 0.001) and FAP (*p* = 0.001), and high numbers of infiltrating CD204^+^ macrophages (*p* = 0.001) (Table 2).

**Table 2:** Expression levels of PAI-1 and LRP1 in human ESCC tissues and their correlation with clinicopathological parameters.

		Expression of PAI-1^a^			Expression of LRP1 (CA)^a^			Expression of LRP1 (ST)^a^		
	Number of cases	Low	High		Low	High		Low	High	
		(*n* = 42)	(*n* = 27)	*p* value	(*n* = 40)	(*n* = 29)	*p* value	(*n* = 27)	(*n* = 42)	*p* value
Age										
<65	32	19	13	N.S.	17	15	N.S.	11	21	N.S.
≥65	37	23	14		23	14		16	21	
Sex										
Male	55	34	21	N.S.	29	26	N.S.	9	5	0.031*
Female	14	8	6		11	3		18	37	
Histological grade^b^										
HGIEN + WDSCC	15	8	7	N.S.	10	5	N.S.	5	10	N.S.
MDSCC + PDSCC	54	34	20		30	24		22	32	
Depth of tumor invasion^b^										
T1	48	37	11	<0.001***	28	20	N.S.	23	25	0.024*
T2 + T3	21	5	16		12	9		4	17	
Lymphatic vessel invasion^b^										
Negative	37	26	11	N.S.	23	14	N.S.	17	20	N.S.
Positive	32	16	16		17	15		10	22	
Blood vessel invasion^b^										
Negative	43	27	16	N.S.	24	19	N.S.	20	23	N.S.
Positive	26	15	11		16	10		7	19	
Lymph node metastasis^b^										
Negative	43	28	15	N.S.	25	18	N.S.	20	23	N.S.
Positive	26	14	12		15	11		7	19	
Stage^c^										
0 + I	38	26	12	N.S.	22	16	N.S.	18	20	N.S.
II + III + IV	31	16	15		18	13		9	22	
Expression of αSMA^d^										
Low	36	29	7	<0.001***	24	12	N.S.	22	14	<0.001***
High	33	13	20		16	17		5	28	
Expression of FAP^d^										
Low	39	31	8	<0.001***	27	12	0.031*	22	17	0.001**
High	30	11	19		13	17		5	25	
Expression of CD163^e^										
Low	34	23	11	N.S.	22	12	N.S.	16	18	N.S.
High	35	19	16		18	17		11	24	
Expression of CD204^e^										
Low	34	27	7	0.002**	24	10	0.036*	20	14	0.001**
High	35	15	20		16	19		7	28	

Data were analyzed by *χ*^*2*^-test; *p* < 0.05 was considered statistically significant: *p < 0.05; **p < 0.01; ***p < 0.001.

^a^The ESCC samples were divided into high and low groups based on the immunoreactivity intensity of PAI-1 in stromal cells or LRP1 in cancer cells (CA) and stromal cells (ST).

^b^According to the Japanese Classification of Esophageal Cancer 10th ed. [47]: HGIEN, high-grade intraepithelial neoplasia; WDSCC, well-differentiated squamous cell carcinoma; MDSCC, moderately differentiated squamous cell carcinoma; PDSCC, poorly differentiated squamous cell carcinoma. T1a, tumor invades mucosa; T1b, tumor invades submucosa; T2, tumor invades muscularis propria; T3, tumor invades adventitia.

^c^According to the TNM classification 7th ed. by UICC [48].

^d^Immunoreactivity around the invasive front of ESCC was divided on the staining area (high: >30%; low: ≤30%).

^e^The median of CD163^+^ or CD204^+^ macrophage counts in cancer nests and stroma within the areas were used to divide the patients into low- and high-groups.

**Fig. 7:**
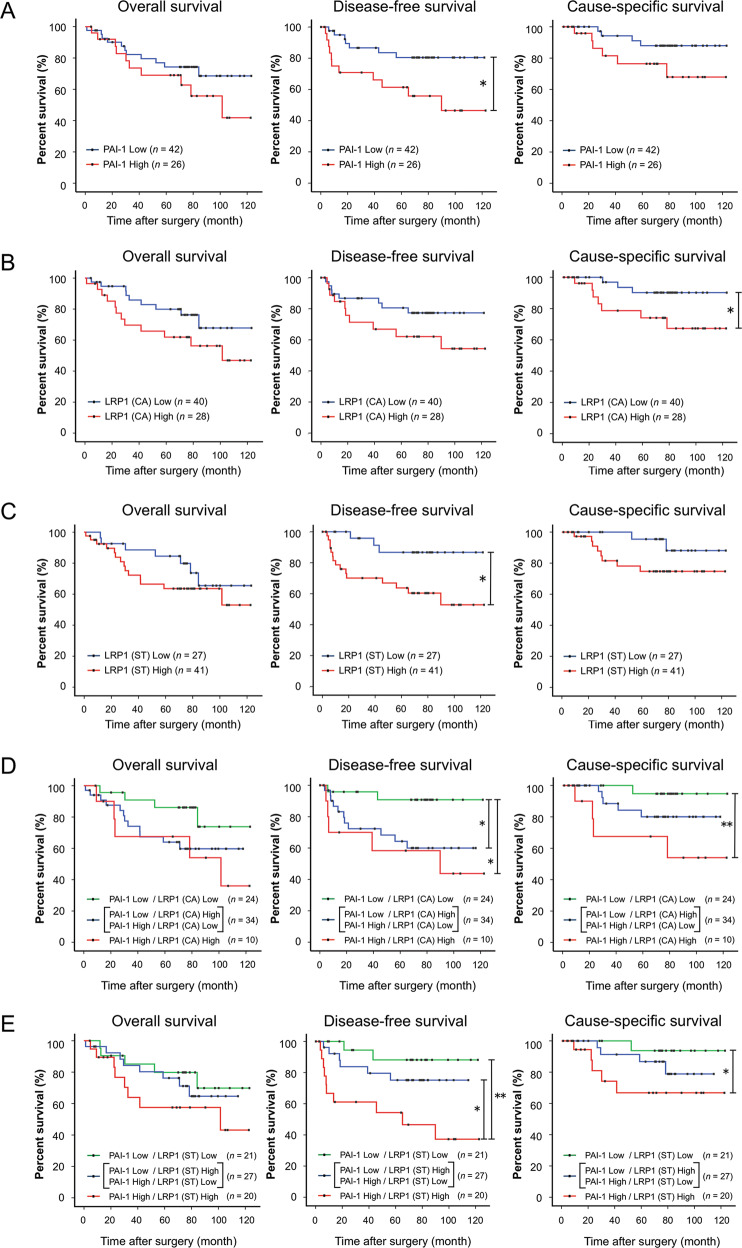
Correlation of PAI-1 and LRP1 expression in esophageal squamous cell carcinoma (ESCC) tissues with the prognosis of ESCC patients. **a** Kaplan–Meier analysis of ESCC patients divided into two groups according to PAI-1 expression: PAI-1 Low group (*n* = 42) and PAI-1 High group (*n* = 26). The log-rank test was performed to determine significance (*p < 0.05). **b** Kaplan–Meier analysis of ESCC patients divided into two groups according to LRP1 expression in cancer nest: LRP1 Low group (*n* = 40) and LRP1 High group (*n* = 28). The log-rank test was performed to determine significance (*p < 0.05). **c** Kaplan–Meier analysis of ESCC patients divided into two groups according to LRP1 expression in the stroma: LRP1 Low group (*n* = 27) and LRP1 High group (*n* = 41). The log-rank test was performed to determine significance (*p < 0.05). **d** Kaplan–Meier analysis of ESCC patients divided into three groups based on the combination of the immunoreactive intensity of PAI-1 in the stroma and LRP1 in cancer nest (CA), as follows: PAI-1 Low/LRP1 (CA) Low (*n* = 24); PAI-1 Low/LRP1 (CA) High and PAI-1 High/LRP1 (CA) Low (*n* = 34); PAI-1 High/LRP1 (CA) High (*n* = 10). The log-rank test was performed to determine significance (*p < 0.05, **p < 0.01). **e** Kaplan–Meier analysis of ESCC *p*atients divided into three groups based on the combination of the immunoreactive intensity of PAI-1 and LRP1 in the stroma (ST), as follows: PAI-1 Low/LRP1 (ST) Low (*n* = 21); PAI-1 Low/LRP1 (ST) High and PAI-1 High/LRP1 (ST) Low (*n* = 27); PAI-1 High/LRP1 (ST) High (*n* = 20). The log-ra*n*k test was performed to determine significance (*p < 0.05, **p < 0.01). CA, cancer nest. ST, stroma.

We next analyzed the prognostic values of PAI-1 and LRP1 expressions using the follow-up data of the 68 patients with ESCC (one patient could not be followed up after surgery). Kaplan–Meier analysis showed that patients with high expression levels of PAI-1 and LRP1 (ST) had significantly shorter disease-free survival (DFS) (*p* = 0.016 and *p* = 0.013, respectively) (Fig. 7a, c), and patients with high expression levels of LRP1 (CA) had significantly shorter CSS (*p* = 0.041) (Fig. 7b). The overall survival of the patients was not significantly affected by PAI-1 (*p* = 0.246), LRP1 (CA) (*p* = 0.128), or LRP1 (ST) (*p* = 0.288) expression. We next divided the patients into three groups based on the combination of PAI-1 and LRP1 (CA) immunoreactivity scores. The patients with high expression levels of both PAI-1 and LRP1 (CA) had significantly shorter DFS (*p* = 0.014) and CSS (*p* = 0.008) compared to those with low expression levels of both PAI-1 and LRP1 (CA) (Fig. 7d). The patients with high expression levels of either PAI-1 or LRP1 (CA) had significantly shorter DFS (*p* = 0.018) compared to those with low expression levels of both PAI-1 and LRP1 (CA) (Fig. 7d). Similarly, we divided the patients into three groups based on the combination of PAI-1 and LRP1 (ST) immunoreactivity scores. The patients with high expression levels of both PAI-1 and LRP1 (ST) had significantly shorter DFS (*p* = 0.003) and CSS (*p* = 0.036) compared to those with low expression levels of both PAI-1 and LRP1 (ST) (Fig. 7e). In addition, the patients with high expression levels of both PAI-1 and LRP1 (ST) had significantly shorter DFS (*p* = 0.027) compared to those with high expression levels of either PAI-1 or LRP1 (ST) (Fig. 7e).

## Discussion

We previously established an indirect coculture assay in which MSCs cocultured with ESCC cells expressed more FAP than monocultured MSCs [13]. We found that FAP was responsible for the tumor-promoting ability and immunosuppressive phenotype of CAFs. Fibroblast heterogeneity has been recognized, but the absence of CAF-specific markers prevents CAF identification and comparison in different studies [18–20]. Costa et al. characterized four CAF subsets using six fibroblast markers identified in primary CAFs isolated from breast cancer specimens [18]. Only the CAF-S1 subset expressed FAP, and CAF-S1 fibroblasts promoted an immunosuppressive microenvironment by secreting CXCL12 and recruiting CD4^+^CD25^+^ T cells. In this study, we confirmed high expression levels of *FAP* (Fig. S2A, B) and *CXCL12* (Fig. S2C) by CAF-like cells. This expression pattern indicates that CAF-like cells established by coculture with ESCC cells are similar to CAF-S1 subset in primary CAFs, and suggests that indirect coculture of MSCs and ESCC cells may be useful for study the tumor-promoting characteristics of CAFs in an ESCC microenvironment.

In this study, we focused on *SERPINE1*, one of the up-regulated genes in CAF-like cells. PAI-1, encoded by *SERPINE1*, is a member of the serpin superfamily and is an essential inhibitor of tissue plasminogen activator and urokinase-type (uPA), activators of plasminogen; PAI-1 can also bind LRP1, a major endocytic receptor [21, 22]. The role of PAI-1 in thrombosis is generally known, and elevated PAI-1 levels in tumor tissues correlate with poor prognosis in patients with certain cancers (e.g., esophageal cancer, head and neck squamous cell carcinoma, gastric adenocarcinoma, pancreatic ductal adenocarcinoma, and urothelial carcinoma) [23–25]. Our findings identified CAFs as an important source of PAI-1 in the ESCC microenvironment. Similar to our results, conditioned medium from colon cancer cells can enhance *SERPINE1* and *ACTA2* expression by CAFs [26]. One study reported that treatment with cisplatin induced CAFs to secrete PAI-1, which contributed to tumor growth and chemoresistance of ESCC cells [27]. However, no reports identify the origin of PAI-1 in the ESCC microenvironment or characterize the detailed effects of CAFs-derived PAI-1 on ESCC cells and macrophages.

Here, we confirmed that PAI-1 accelerated the migration and invasion of ESCC cells and macrophages by activating Akt and Erk1/2 signaling pathways in a paracrine manner. Tumor cell-derived PAI-1 enhances the progression of some cancers including ESCC, head and neck cancer, and breast cancer [27–31]. In addition, PAI-1 secreted by non-small cell lung cancer and fibrosarcoma can induce monocyte migration [32]; however, how CAF-derived PAI-1 promotes ESCC progression remains unclear. The main findings of this study suggest that PAI-1 secreted by CAFs contributes to the migration, invasion, and intracellular signaling of ESCC cells and macrophages in a paracrine manner. Recent in vivo studies have noted that PAI-1 inhibitors suppress tumor growth, angiogenesis, and metastasis of some cancers (e.g., ovarian cancer, lung carcinoma, urothelial carcinoma, and fibrosarcoma) [33–36]. These findings suggest that targeting PAI-1 is a potential therapeutic strategy for patients with ESCC.

Our in vitro results suggest that the PAI-1/LRP1 axis is essential to promote the migration and invasion of ESCC cells and macrophages. Several studies have reported that the binding of PAI-1 to LRP1 is involved in the migration, invasion, and Akt signaling [21, 23, 29, 32, 37, 38]. PAI-1 can interact with not only LRP1 but also uPA, which binds the uPA plasminogen activator receptor (uPAR). The uPA/uPAR/PAI-1 system stimulates tumor vascularization by promoting the migration of endothelial cells [23]. However, we confirmed that TE-series ESCC cells did not express uPAR (Fig. S8).

Another group has reported that PAI-1 promotes M2 polarization of monocytes via an IL6/STAT3 autocrine loop in fibrosarcoma [32]; however, we did not observe M2 polarization following rhPAI-1 treatment (Fig. S7). This discrepancy may be due to different experimental models; the study above evaluated the effects of PAI-1 on the shift of PBMos toward the M2 phenotype, while we used PBMos treated with M-CSF, which we defined as macrophages. M-CSF can induce M2 polarization of monocytes [39–42]. This may be why rhPAI-1 did not increase the expression of CD163 and CD204 in macrophages in our study. A previous study reported that macrophages expressed LRP1 [37], and in the present study, we confirmed that CD204^+^ macrophages in human ESCC tissue, TAMs, expressed LRP1 by double immunofluorescence (Fig. 6e), and that the expression level of PAI-1 in stromal cells was associated with high numbers of infiltrating CD204^+^ macrophages. These results indicate that CAF-derived PAI-1 may promote TAM recruitment to ESCC tissue.

Masuda et al. showed that stromal cells express higher levels of PAI-1 than cancer cells in lung adenocarcinoma tissues and that PAI-1 expression correlated with αSMA levels and depth of tumor invasion [43]. These findings are consistent with our results. Recent studies suggest that elevated PAI-1 and LRP1 expression are indicators of poor prognosis for patients with various cancers [23–25, 33, 38, 44, 45]; however, few studies have distinguished between the expression levels of PAI-1 by cancer nests and tumor stroma [27, 46]. Consistent with our findings, increased expression of PAI-1 by the tumor stroma correlates with poor prognosis in ESCC [27] and breast cancer patients [46], but no reports have evaluated the immunohistochemical expression intensity of both PAI-1 and LRP1 in tumor tissues. Our data demonstrate that ESCC patients with concurrently high expression of PAI-1 by stromal cells and of LRP1 by either cancer cells or stromal cells including TAMs, or both have the shortest survival duration. This finding may support our in vitro results that identify the contribution of the PAI-1/LRP1 axis in ESCC progression.

In conclusion, we demonstrated that PAI-1 derived from CAFs promoted the migration and invasion of both ESCC cells and macrophages *via* phosphorylation of Akt and Erk1/2 through interaction with LRP1. PAI-1 in CAFs is a potential prognostic factor for patients with ESCC. Targeting the PAI-1/LRP1 axis might, therefore, be a therapeutic approach for ESCC.

## Supplementary information

Figure S1_S2_S3_S4_S5_S6_S7_S8 and Table S1_S2_S3_S4
