# Supplementary material for: PAI-1 derived from cancer-associated fibroblasts in esophageal squamous cell carcinoma promotes the invasion of cancer cells and the migration of macrophages
Source: Lab Invest. 2020 Dec 12;101(3):353–68. doi: 10.1038/s41374-020-00512-2 (PMC7892342; doi:10.1038/s41374-020-00512-2)
Supplement: Supplementary file 1 — Figure S1_S2_S3_S4_S5_S6_S7_S8 and Table S1_S2_S3_S4 [file 41374_2020_512_MOESM1_ESM.pdf]

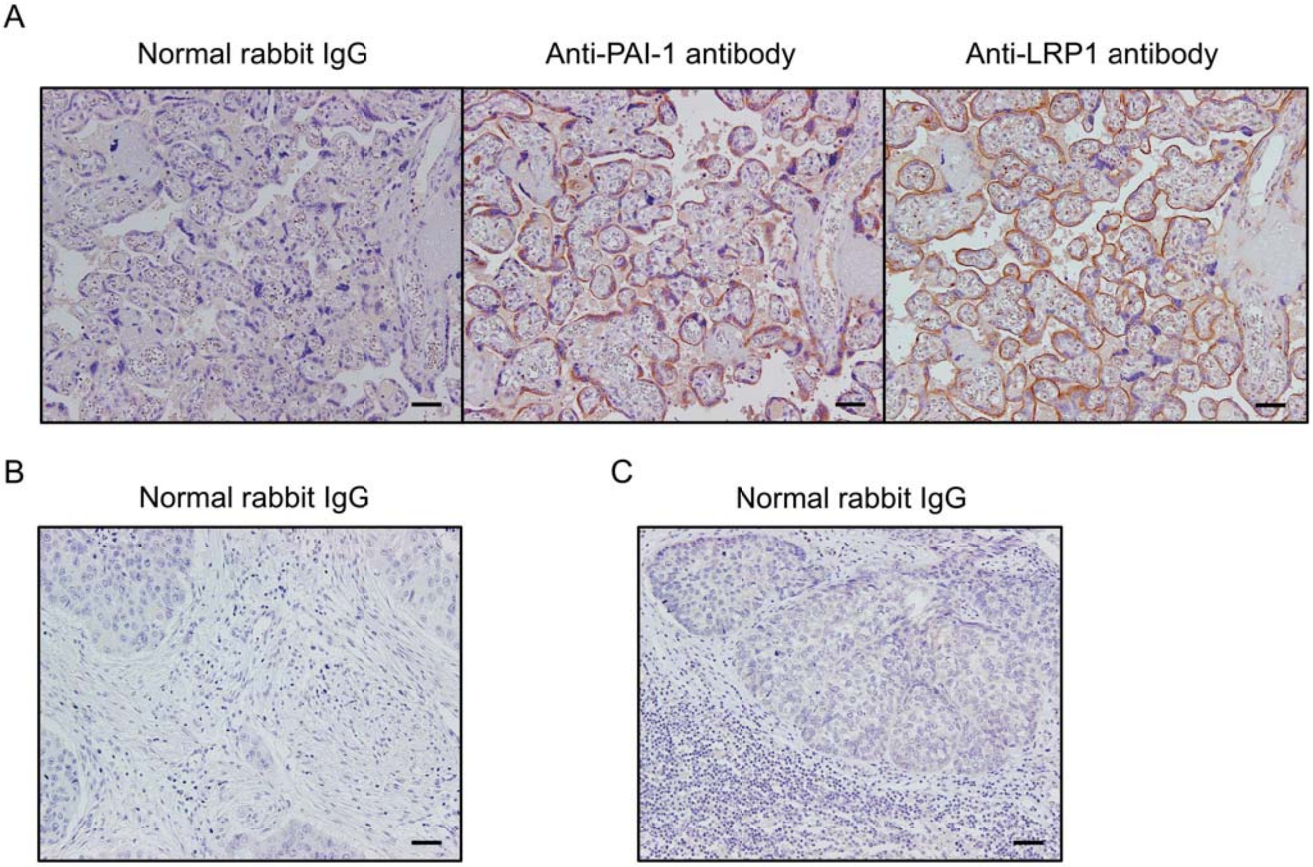

Fig. S1. A: Immunohistochemical staining in placental tissues using normal rabbit IgG, anti-PAI-1 antibody, and anti-LRP1 antibody. **B:** Immunohistochemical staining in human ESCC tissues using control rabbit IgG under the same condition as anti-PAI-1 antibody. **C:** Immunohistochemical staining in human ESCC tissues using control rabbit IgG under the same condition as anti-LRP1 antibody. Scale bars = 100 μm.

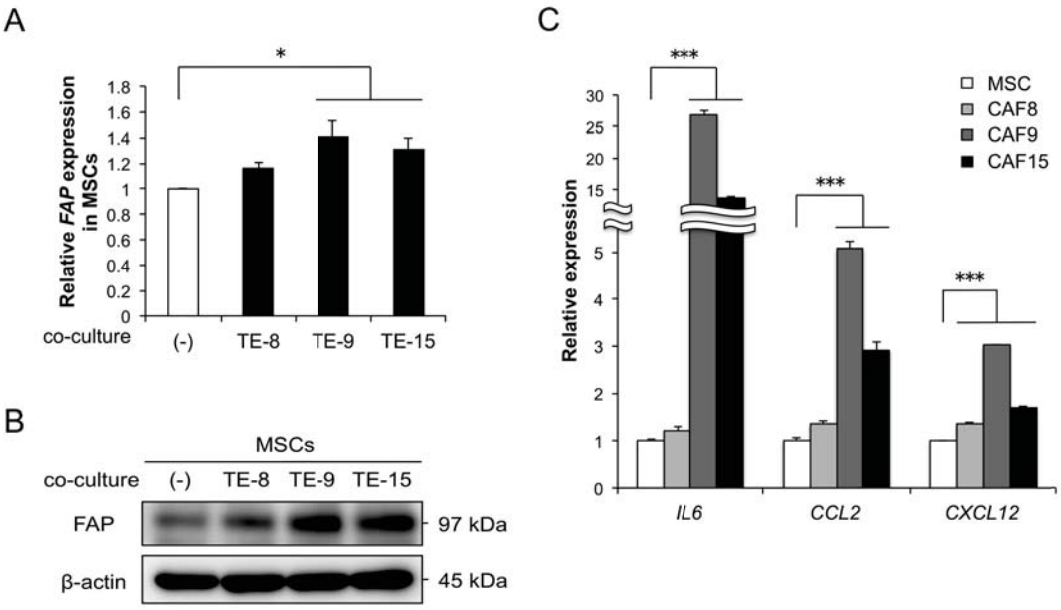

Fig. S2. The expression levels of *FAP*, *IL6*, *CCL2,* and *CXCL12* in MSCs and CAF-like cells.

A: The expression levels of *FAP* mRNA in MSCs monocultured or co-cultured with ESCC cells were determined by quantitative RT-PCR, normalized against *ACTB* (*β-actin*) expression. Data are the means ± SEM of triplicate wells and are representative of three independent experiments (*$p < 0.05$). **B:** The expression levels of FAP in MSCs monocultured or co-cultured with ESCC cells were confirmed by western blotting. Anti-FAP and β-actin antibodies were used. **C:** The levels of *IL6*, *CCL2,* and *CXCL12* mRNA in MSCs and CAF-like cells were determined by quantitative RT-PCR, normalized against *ACTB* (*β-actin*) expression. Data are the means ± SEM of triplicate wells and are representative of three independent experiments (***$p < 0.001$).

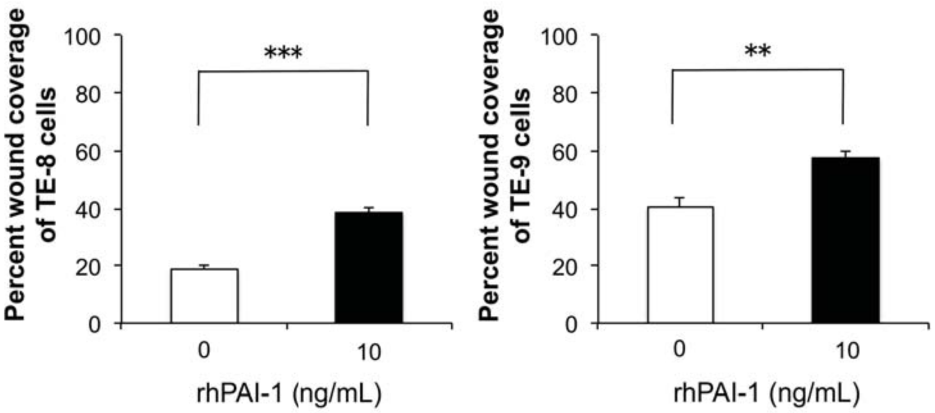

Fig. S3. The effects of recombinant human (rh) PAI-1 on TE-8 and TE-9 cells using wound healing assay. Wound coverage were calculated in four randomly chosen fields. Data are the means ± SEM of triplicate wells and are representative of three independent experiments. $^{**}p < 0.01$, $^{***}p < 0.001$.

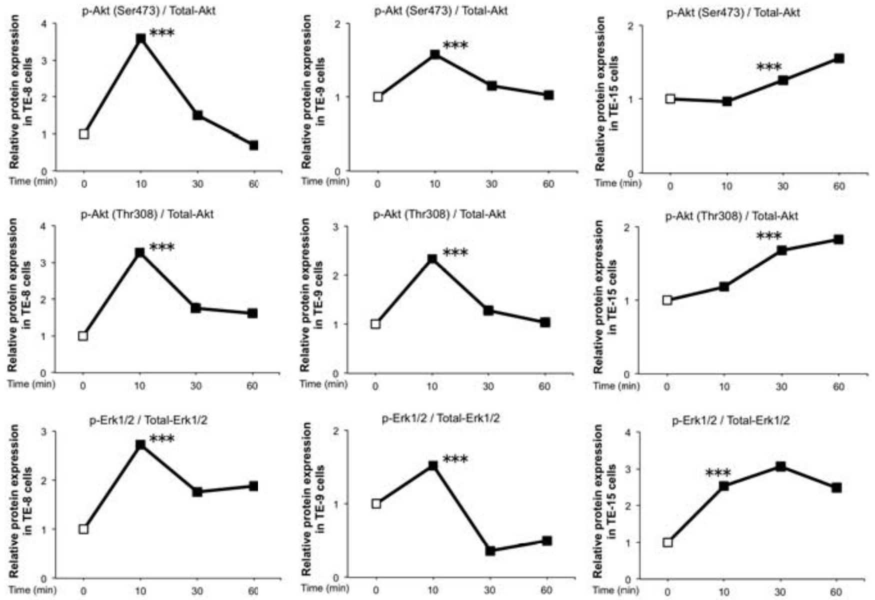

Fig. S4. Densitometric analysis of western blotting bands (Figure 3E) was performed with ImageJ (National Institutes of Health, Maryland, USA). Statistical significance at 10 or 30 min against 0 min were determined by two-tailed Student's *t*-test. The results are mean ± SEM. $***p < 0.001$.

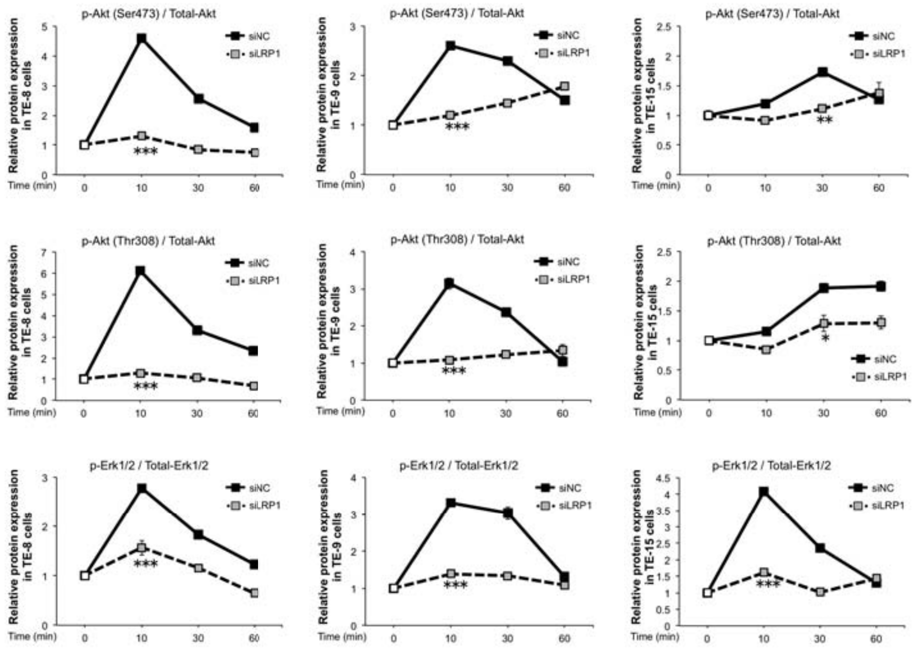

Fig. S5. Densitometric analysis of western blotting bands (Figure 4F) was performed with ImageJ (National Institutes of Health, Maryland, USA). Statistical significance between siNC and siLRP1 at same time (10 or 30 min) were determined by two-tailed Student's *t*-test. The results are mean ± SEM. *$p < 0.05$, **$p < 0.01$, ***$p < 0.001$.

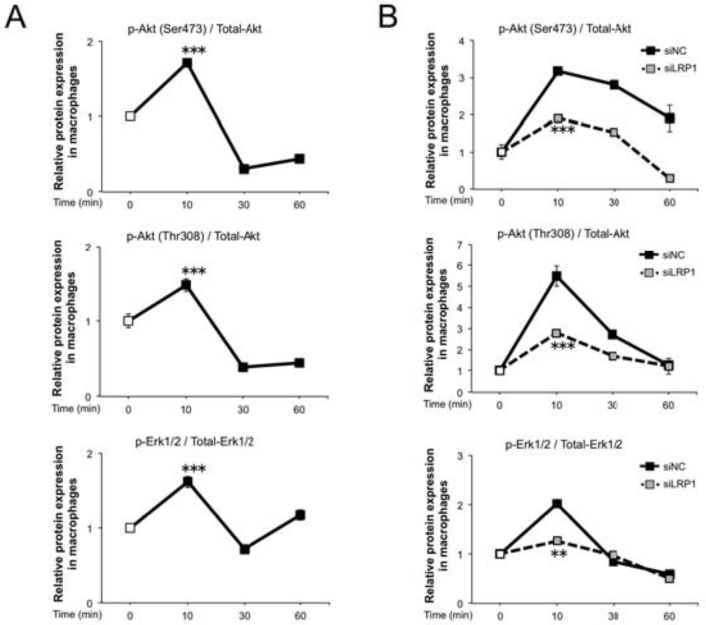

Fig. S6. A: Densitometric analysis of western blotting bands (Figure 5I) was performed with ImageJ (National Institutes of Health, Maryland, USA). Statistical significance at 10 or 30 min against 0 min were determined by two-tailed Student's *t*-test. The results are mean ± SEM. ***$p < 0.001$. **B:** Densitometric analysis of western blotting bands (Figure 5P) was performed with ImageJ. Statistical significance between siNC and siLRP1 at 10 min were determined by two-tailed Student's *t*-test. The results are mean ± SEM. **$p < 0.01$, ***$p < 0.001$.

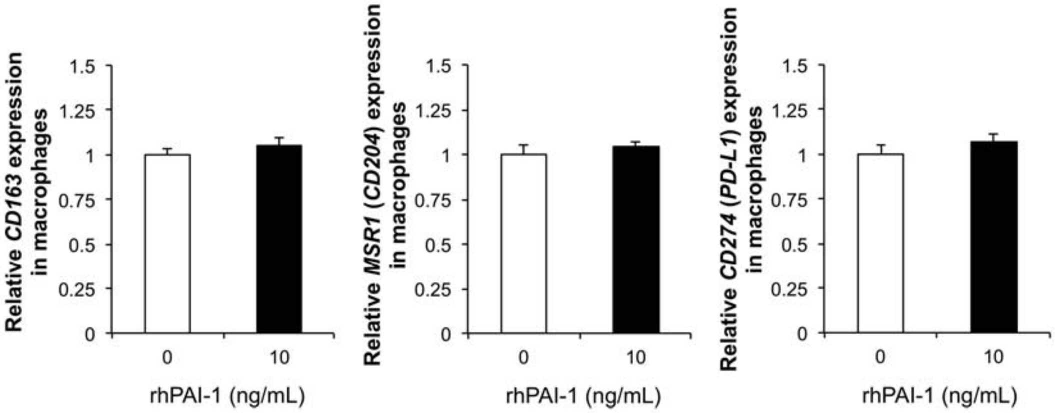

Fig. S7. The levels of *CD163*, *MSR1* (*CD204*), and *CD274* (*PD-L1*) mRNA in the macrophages treated with recombinant human PAI-1 for 6 days were determined by quantitative RT-PCR, normalized against *ACTB* (*β-actin*) expression. Data are the means ± SEM of triplicate wells and are representative of three independent experiments.

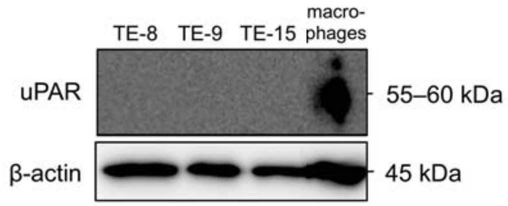

Fig. S8. The expression levels of uPAR in the ESCC cells and macrophages were confirmed by western blotting. Anti-uPAR and β-actin antibodies were used.

Supplementary Table S1

The primer sets used for RT-PCR and the probes for quantitative RT-PCR

Gene		Primer sequences
LRP1	Forward:	5’- AGA AGT AGC AGG ACC AGA GGG -3’
	Reverse:	5’- TCA GTA CCC AGG CAG TTA TGC -3’
GAPDH	Forward:	5’- ACC ACA GTC CAT GCC ATC AC -3’
	Reverse:	5’- TCC ACC ACC CTG TTG CTG TA -3’

Gene	Probe product code[a]
FAP	Hs00990806_m1
SERPINE1	Hs00167155_m1
IL6	Hs00174131_m1
CCL2	Hs00234140_m1
CXCL12	Hs00171022_m1
CD163	Hs00174705_m1
MSR1	Hs00234007_m1
CD274	Hs00204257_m1
ACTB	Hs01060665_g1

[a]The probes for those genes were from TaqMan Gene Expression Assays (Applied Biosystems).

Supplementary Table S2 Primary and secondary antibodies used for experiments

Primary antibody	Host	Clonality (Clone)	Product code	Company	Application[a]
Anti-FAP	Sheep	Polyclonal	AF3715	R&D Systems, Minneapolis, MN	WB, IHC
Anti-LRP1	Rabbit	Monoclonal (EPR3724)	ab92544	Abcam, Cambridge, UK	WB, IHC, IF
Anti-PAI-1	Rabbit	Polyclonal	ab66705	Abcam	IHC
Anti-αSMA	Mouse	Monoclonal (1A4)	A5228	Sigma-Aldrich, St. Louis, MO	IHC
Anti-CD163	Mouse	Monoclonal (10D6)	NCL-CD163	Leica Biosystems, Newcastle, UK	IHC
Anti-CD204	Mouse	Monoclonal (SRA-E5)	KT022	Trans Genic, Kobe, Japan	IHC, IF
Normal rabbit IgG	Rabbit	Polyclonal	sc-2027	Santa Cruz Biotechnology, Dallas, TX	IHC
Anti-β-actin	Rabbit	Polyclonal	# 4970	Cell Signaling Technology, Beverly, MA	WB
Anti-uPAR	Mouse	Monoclonal (E-3)	sc-376494	Santa Cruz Biotechnology	WB
Anti-Akt	Rabbit	Polyclonal	# 9272	Cell Signaling Technology	WB
Anti-Akt (phosphorylation of Ser473)	Rabbit	Monoclonal (D9E)	# 4060	Cell Signaling Technology	WB
Anti-Akt (phosphorylation of Thr308)	Rabbit	Monoclonal (C31E5E)	# 2965	Cell Signaling Technology	WB
Anti-p44/42 MAPK (Erk1/2)	Rabbit	Polyclonal	# 9102	Cell Signaling Technology	WB
Anti-Erk1/2 (phosphorylation of Thr202/Tyr204)	Rabbit	Polyclonal	# 9101	Cell Signaling Technology	WB

Secondary antibody	Host	Conjugate	Product code	Company	Application[a]
Anti-sheep Ig[b]	Donkey	HRP	sc-2473	Santa Cruz Biotechnology	WB
Anti-mouse Ig[b]	Sheep	HRP	NA931V	GE Healthcare, NSW, Australia	WB
Anti-rabbit Ig[b]	Donkey	HRP	NA934V	GE Healthcare	WB
Anti-rabbit Ig[b]	Donkey	Alexa Fluor-488	711-545-152	Jackson ImmunoResearch Laboratories, West Grove, PA	IF
Anti-mouse Ig[b]	Donkey	Cy3	715-165-150	Jackson ImmunoResearch Laboratories	IF

[a]WB, Western blotting; IHC, immunohistochemistry for formalin-fixed paraffin embedded sections; IF, immunofluorescence

for formalin-fixed paraffin embedded sections

[b]Ig, immunoglobulin

Supplementary Table S3. Patient characteristics

Characteristics	*n*	(%)
Age		
< 65	32	(46.4)
≥ 65	37	(53.6)
Sex		
Male	55	(79.7)
Female	14	(20.3)
Histological grade[a]		
HGIEN	4	(5.8)
WDSCC	11	(15.9)
MDSCC	43	(62.3)
PDSCC	11	(15.9)
Depth of tumor invasion[a]		
T1a	18	(26.1)
T1b	30	(43.5)
T2	6	(8.7)
T3	15	(21.7)
Lymphatic vessel invasion[a]		
ly0	37	(53.6)
ly1	20	(29.0)
ly2	8	(11.6)
ly3	4	(5.8)
Blood vessel invasion[a]		
v0	43	(62.3)
v1	19	(27.5)
v2	5	(7.2)
v3	2	(2.9)
Lymph node metastasis[a]		
N0	43	(62.3)
N1	13	(18.8)
N2	11	(15.9)
N3	2	(2.9)
Distant metastasis[a]		
M0	67	(97.1)
M1	2	(2.9)
Residual tumor[a]		
R0	62	(89.9)
R1	4	(5.8)

R2	3	(4.3)
Stage[b]		
0	4	(5.8)
I	34	(49.3)
II	17	(24.6)
III	12	(17.4)
IV	2	(2.2)

[a]According to the Japanese Classification of Esophageal Cancer 10th ed. [47]: HGIEN, high-grade intraepithelial neoplasia; WDSCC, well-differentiated squamous cell carcinoma; MDSCC, moderately differentiated squamous cell carcinoma; PDSCC, poorly differentiated squamous cell carcinoma. T1a, tumor invades mucosa; T1b, tumor invades submucosa; T2, tumor invades muscularis propria; T3, tumor invades adventitia.

[b]According to the TNM classification 7th ed. by UICC [48].

Supplementary Table S4 Expression profile of up-regulated and down-regulated genes in CAF-like cells; the genes were selected by an expression ratio difference of > 2.0 or < 0.5 in CAF9

Accession number	Gene title	Symbol	Ratio		
			CAF8	CAF9	CAF15
XM_005250340.3	leptin	*LEP*	15.90	55.22	25.49
NM_001024466.1	superoxide dismutase 2, mitochondrial	*SOD2*	1.68	25.71	15.26
NR_104213.1	angiopoietin like 4	*ANGPTL4*	16.43	24.25	14.23
NM_002982.3	C-C motif chemokine ligand 2	*CCL2*	2.26	23.81	22.54
NM_000064.3	complement component 3	*C3*	2.65	14.84	12.04
NM_001144936.1	chromosome 11 open reading frame 95	*C11orf95*	11.92	12.73	13.18
NM_018476.3	brain expressed X-linked 1	*BEX1*	3.58	12.59	8.70
XM_011515390.1	interleukin 6	*IL6*	1.09	11.80	9.84
NM_001318914.1	interleukin 1 receptor antagonist	*IL1RN*	9.44	9.77	12.05
NM_001172173.1	cysteine-serine-rich nuclear protein 3	*CSRNP3*	9.94	9.10	8.88
NM_030754.4	serum amyloid A2	*SAA2*	3.67	8.83	7.47
NM_001293643.1	placental growth factor	*PGF*	8.10	7.86	10.50
NM_173647.3	ring finger protein 149	*RNF149*	6.23	7.35	7.49
NM_015444.2	transmembrane protein 158 (gene/pseudogene)	*TMEM158*	1.91	6.57	3.78
NM_003897.3	immediate early response 3	*IER3*	2.11	6.52	4.78
NM_001293642.1	carbonic anhydrase XII	*CA12*	2.21	6.25	4.37
NM_001185056.1	claudin 11	*CLDN11*	2.34	6.20	5.80
XM_006717019.1	FK506 binding protein 15	*FKBP15*	5.33	6.12	5.41
NM_002759.3	eukaryotic translation initiation factor 2 alpha kinase 2	*EIF2AK2*	3.64	6.05	3.84
NM_005951.2	metallothionein 1H	*MT1H*	1.05	5.73	3.63
XR_931600.1	uncharacterized LOC105369633	*LOC105369633*	3.20	5.54	3.37
NM_001005353.2	adenylate kinase 4	*AK4*	1.97	5.42	3.29
NM_005947.2	metallothionein 1B	*MT1B*	1.25	5.26	3.49
NM_001276687.1	metallothionein 1H-like 1	*MT1HL1*	1.28	5.01	3.47
XM_005250901.3	kelch like family member 38	*KLHL38*	4.63	4.97	4.66
NM_005953.3	metallothionein 2A	*MT2A*	1.57	4.83	3.85
NM_005952.3	metallothionein 1X	*MT1X*	1.21	4.78	2.86
NM_002130.7	3-hydroxy-3-methylglutaryl-CoA synthase 1	*HMGCS1*	3.77	4.60	3.02

Accession number	Gene title	Symbol	Ratio		
			CAF8	CAF9	CAF15
NM_000710.3	bradykinin receptor B1	*BDKRB1*	1.22	4.60	3.36
XM_006718750.1	leupaxin	*LPXN*	2.27	4.59	4.39
NM_004335.3	bone marrow stromal cell antigen 2	*BST2*	2.31	4.47	2.34
NM_020529.2	NFKB inhibitor alpha	*NFKBIA*	1.23	4.42	2.81
NM_000602.4	serpin peptidase inhibitor, clade E (nexin, plasminogen activator inhibitor type 1), member 1	*SERPINE1*	1.43	4.37	3.62
XM_005269005.2	DNA damage regulated autophagy modulator 1	*DRAM1*	1.05	4.35	3.47
XM_006718205.1	calcium release activated channel regulator 2B	*CRACR2B*	4.53	4.34	4.82
NM_013332.3	hypoxia inducible lipid droplet associated	*HILPDA*	1.59	4.05	2.16
NM_001271004.1	tissue factor pathway inhibitor 2	*TFPI2*	1.58	4.04	3.31
NM_153833.2	H1 histone family member O, oocyte specific	*H1FOO*	0.99	4.03	2.36
NM_001313945.1	ras homolog family member A	*RHOA*	4.24	3.98	4.18
XM_006711036.1	dehydrogenase/reductase (SDR family) member 3	*DHRS3*	5.76	3.78	6.20
NM_001336.3	cathepsin Z	*CTSZ*	3.35	3.76	2.05
NM_001012456.1	Sec61 translocon gamma subunit	*SEC61G*	3.06	3.61	2.78
NM_004052.3	BCL2/adenovirus E1B 19kDa interacting protein 3	*BNIP3*	1.63	3.58	2.85
XM_011524556.1	aldolase, fructose-bisphosphate C	*ALDOC*	2.47	3.22	2.11
NM_022873.2	interferon, alpha-inducible protein 6	*IFI6*	1.27	3.10	1.07
XM_011542748.1	cysteine and histidine rich domain containing 1	*CHORDC1*	2.85	3.09	3.18
NM_014367.3	family with sequence similarity 162 member A	*FAM162A*	1.71	3.09	1.94
XM_011519493.1	6-phosphofructo-2-kinase/fructose-2,6-biphosphatase 3	*PFKFB3*	2.01	3.05	2.23
NM_002006.4	fibroblast growth factor 2	*FGF2*	0.97	3.05	1.54
XM_005252467.2	phosphofructokinase, platelet	*PFKP*	2.03	3.00	2.45
NM_205858.1	neuromedin B	*NMB*	1.09	2.98	1.83
XM_011524556.1	aldolase, fructose-bisphosphate C	*ALDOC*	2.36	2.91	2.00
NM_001935.3	dipeptidyl peptidase 4	*DPP4*	0.98	2.91	2.48
NM_005101.3	ISG15 ubiquitin-like modifier	*ISG15*	1.08	2.88	0.85
NM_001270533.1	Wilms tumor 1 associated protein	*WTAP*	1.09	2.88	2.34
XM_011536684.1	endoplasmic reticulum oxidoreductase alpha	*ERO1A*	1.82	2.86	1.96
NM_002228.3	jun proto-oncogene	*JUN*	1.44	2.83	2.18
NM_001162371.2	uncharacterized LOC728392	*LOC728392*	1.30	2.83	2.06
NM_002506.2	nerve growth factor (beta polypeptide)	*NGF*	1.45	2.77	2.43

Accession number	Gene title	Symbol	Ratio		
			CAF8	CAF9	CAF15
NM_001124.2	adrenomedullin	*ADM*	1.11	2.77	2.04
NM_014391.2	ankyrin repeat domain 1	*ANKRD1*	1.68	2.77	1.93
NM_013300.2	family with sequence similarity 216 member A	*FAM216A*	2.92	2.76	2.28
XM_005252186.3	UDP-glucose ceramide glucosyltransferase	*UGCG*	1.44	2.76	2.51
NM_005257.5	GATA binding protein 6	*GATA6*	2.11	2.64	3.09
NM_020197.2	SET and MYND domain containing 2	*SMYD2*	2.52	2.62	2.02
XR_429442.1	lactamase beta	*LACTB*	1.58	2.58	2.06
NM_032947.4	small integral membrane protein 3	*SMIM3*	2.51	2.58	2.59
NM_001077516.1	solute carrier family 39 member 7	*SLC39A7*	2.01	2.55	1.78
NM_000189.4	hexokinase 2	*HK2*	2.15	2.54	1.51
NM_001165252.1	keratin associated protein 2-3	*KRTAP2-3*	1.98	2.53	2.70
NM_007115.3	TNF alpha induced protein 6	*TNFAIP6*	0.65	2.53	2.43
NM_000935.2	procollagen-lysine, 2-oxoglutarate 5-dioxygenase 2	*PLOD2*	1.73	2.52	2.36
NM_000090.3	collagen type III alpha 1	*COL3A1*	1.27	2.51	1.98
NM_001204813.1	5'-nucleotidase ecto	*NT5E*	1.27	2.48	1.58
NM_006516.2	solute carrier family 2 member 1	*SLC2A1*	2.21	2.46	1.83
XM_011544825.1	cytochrome b561 family member A3	*CYB561A3*	1.29	2.44	2.44
XM_011536684.1	endoplasmic reticulum oxidoreductase alpha	*ERO1A*	1.66	2.43	1.73
NR_038911.1	MIF antisense RNA 1	*MIF-AS1*	1.70	2.39	2.01
NM_001856.3	collagen type XVI alpha 1	*COL16A1*	0.73	2.37	1.68
NM_001159287.1	triosephosphate isomerase 1	*TPI1*	1.92	2.36	2.27
NM_001316320.1	procollagen-lysine, 2-oxoglutarate 5-dioxygenase 1	*PLOD1*	1.76	2.33	2.09
NM_018004.2	transmembrane protein 45A	*TMEM45A*	1.40	2.31	1.66
XM_006719552.2	CAP-Gly domain containing linker protein 1	*CLIP1*	2.35	2.28	2.23
NM_001402.5	eukaryotic translation elongation factor 1 alpha 1	*EEF1A1*	1.93	2.27	2.29
NM_001033886.2	C-X-C motif chemokine ligand 12	*CXCL12*	2.58	2.27	2.48
NM_031957.1	keratin associated protein 1-5	*KRTAP1-5*	1.90	2.27	2.51
NR_133921.1	frizzled class receptor 6	*FZD6*	2.29	2.26	2.28
NM_004477.2	FSHD region gene 1	*FRG1*	1.84	2.24	2.16
NR_109934.1	KIAA0101	*KIAA0101*	1.75	2.23	1.60
NM_001300965.1	cysteine and glycine rich protein 2	*CSRP2*	2.48	2.18	1.54
NM_001163446.1	carboxypeptidase A4	*CPA4*	1.66	2.18	2.51

Accession number	Gene title	Symbol	Ratio		
			CAF8	CAF9	CAF15
NM_002318.2	lysyl oxidase like 2	*LOXL2*	1.37	2.17	1.77
NM_002546.3	tumor necrosis factor receptor superfamily member 11b	*TNFRSF11B*	1.85	2.17	2.30
NM_001136016.3	amyloid beta precursor protein	*APP*	2.57	2.14	1.55
NM_178507.2	out at first homolog	*OAF*	1.89	2.13	1.87
XM_011516792.1	N-myc downstream regulated 1	*NDRG1*	1.38	2.13	1.47
NM_001288757.1	BRISC and BRCA1 A complex member 1	*BABAM1*	2.29	2.11	2.12
XM_005249543.1	insulin induced gene 1	*INSIG1*	1.23	2.10	1.33
XM_005254301.1	gremlin 1, DAN family BMP antagonist	*GREM1*	1.58	2.09	1.54
NM_006806.4	BTG family member 3	*BTG3*	1.36	2.09	1.67
NM_017459.2	microfibrillar associated protein 2	*MFAP2*	3.10	2.08	1.96
NM_016588.2	neuritin 1	*NRN1*	1.05	2.08	1.36
XM_011525100.1	FK506 binding protein 10	*FKBP10*	2.06	2.07	1.84
NM_001318941.1	tissue factor pathway inhibitor	*TFPI*	1.44	2.05	2.11
NM_001291995.1	serum/glucocorticoid regulated kinase 1	*SGK1*	1.38	2.04	1.76
XM_005266412.1	polymerase (RNA) I subunit D	*POLR1D*	1.53	2.03	1.61
NM_002852.3	pentraxin 3	*PTX3*	0.62	2.03	2.01
NM_032849.3	mesenteric estrogen-dependent adipogenesis	*MEDAG*	1.78	2.03	2.24
NM_000158.3	glucan (1,4-alpha-), branching enzyme 1	*GBE1*	1.20	2.03	1.65
NM_001142595.1	prolyl 4-hydroxylase subunit alpha 1	*P4HA1*	1.98	2.01	1.85

Accession number	Gene title	Symbol	Ratio		
			CAF8	CAF9	CAF15
NM_002009.3	fibroblast growth factor 7	*FGF7*	0.76	0.11	0.17
NM_006855.3	KDEL endoplasmic reticulum protein retention receptor 3	*KDELR3*	0.72	0.12	0.85
NM_003713.4	phospholipid phosphatase 3	*PLPP3*	0.73	0.16	0.29
NM_001012973.1	placenta specific 9	*PLAC9*	0.65	0.27	0.36
NM_054027.4	ANKH inorganic pyrophosphate transport regulator	*ANKH*	0.76	0.27	0.42
XM_006716587.1	ectonucleotide pyrophosphatase/phosphodiesterase 2	*ENPP2*	0.35	0.27	0.43
XR_243204.1	hyaluronan and proteoglycan link protein 3	*HAPLN3*	0.47	0.29	0.29
NM_001293178.1	protease, serine 23	*PRSS23*	0.79	0.30	0.32
NM_015151.3	disco interacting protein 2 homolog A	*DIP2A*	0.35	0.30	0.32
NM_000954.5	prostaglandin D2 synthase	*PTGDS*	0.54	0.31	0.33
NM_001253908.1	aldo-keto reductase family 1, member C3	*AKR1C3*	0.68	0.31	0.37
XM_011510446.1	zinc finger CCCH domain-containing protein 18-like	*LOC101060391*	0.35	0.31	0.31
NM_000396.3	cathepsin K	*CTSK*	0.51	0.31	0.19
XM_011538693.1	SWI/SNF related, matrix associated, actin dependent regulator of chromatin, subfamily c, member 2	*SMARCC2*	0.32	0.32	0.33
NM_001408.2	cadherin EGF LAG seven-pass G-type receptor 2	*CELSR2*	0.38	0.32	0.36
XR_928319.1	angiopoietin 1	*ANGPT1*	0.84	0.32	0.40
NG_031992.1	wingless-type MMTV integration site family member 5A	*WNT5A*	0.32	0.32	0.32
XM_005244843.2	influenza virus NS1A binding protein	*IVNS1ABP*	0.72	0.33	0.32
NR_123733.1	microfibrillar associated protein 5	*MFAP5*	0.81	0.33	0.42
XM_011516432.1	striatin interacting protein 2	*STRIP2*	0.41	0.34	0.38
XR_929742.1	RAB GTPase activating protein 1	*RABGAP1*	0.70	0.34	0.41
NM_002775.4	HtrA serine peptidase 1	*HTRA1*	0.82	0.34	0.66
NM_020190.3	olfactomedin like 3	*OLFML3*	0.82	0.35	0.42
NM_004503.3	homeobox C6	*HOXC6*	0.42	0.35	0.38
NR_104411.1	solute carrier family 25 member 44	*SLC25A44*	0.42	0.35	0.46
NM_005398.5	protein phosphatase 1 regulatory subunit 3C	*PPP1R3C*	1.05	0.35	0.46
XM_011517895.1	natriuretic peptide receptor 2	*NPR2*	0.43	0.35	0.41
XM_011521744.1	zinc finger protein 770	*ZNF770*	0.38	0.35	0.36
NM_002983.2	C-C motif chemokine ligand 3	*CCL3*	0.40	0.35	0.36
NM_001282582.1	matrix-remodelling associated 8	*MXRA8*	0.42	0.36	0.50
NM_001286137.1	NAD(P)H dehydrogenase, quinone 1	*NQO1*	0.63	0.36	0.54

Accession number	Gene title	Symbol	Ratio		
			CAF8	CAF9	CAF15
NM_144976.3	zinc finger protein 564	*ZNF564*	0.39	0.36	0.30
NM_001015056.1	rhotekin	*RTKN*	0.35	0.36	0.35
NM_006486.2	fibulin 1	*FBLN1*	0.96	0.37	0.71
NM_033256.2	protein phosphatase 1 regulatory inhibitor subunit 14A	*PPP1R14A*	1.13	0.37	0.38
XM_011522217.1	solute carrier family 28 member 1	*SLC28A1*	0.41	0.38	0.37
NM_058179.3	phosphoserine aminotransferase 1	*PSAT1*	0.55	0.38	0.48
NR_024047.1	wingless-type MMTV integration site family member 2	*WNT2*	0.43	0.38	0.38
NM_001145033.1	chromosome 11 open reading frame 96	*C11orf96*	0.40	0.38	0.33
NM_002874.4	RAD23 homolog B, nucleotide excision repair protein	*RAD23B*	0.77	0.39	0.56
XM_011520181.1	PHD finger protein 21A	*PHF21A*	0.39	0.39	0.38
XM_011509141.1	family with sequence similarity 129 member A	*FAM129A*	0.66	0.39	0.41
XM_006716284.1	clusterin	*CLU*	0.77	0.39	0.54
NM_004489.4	G protein pathway suppressor 2	*GPS2*	0.50	0.39	0.49
NM_001884.3	hyaluronan and proteoglycan link protein 1	*HAPLN1*	0.92	0.39	0.41
NM_178354.2	late cornified envelope 1F	*LCE1F*	0.39	0.39	0.37
NM_006522.3	wingless-type MMTV integration site family member 6	*WNT6*	0.41	0.39	0.43
NM_024310.4	pleckstrin homology and FYVE domain containing 1	*PLEKHF1*	0.66	0.39	0.46
NM_001301188.1	tribbles pseudokinase 3	*TRIB3*	0.49	0.40	0.50
NM_001282582.1	matrix-remodelling associated 8	*MXRA8*	0.62	0.40	0.46
XM_011542609.1	crystallin alpha B	*CRYAB*	0.61	0.40	0.42
NM_001304388.1	golgin A6 family-like 2	*GOLGA6L2*	0.40	0.40	0.40
XM_011538794.1	glycosyltransferase 8 domain containing 2	*GLT8D2*	0.90	0.40	0.52
NM_020190.3	olfactomedin like 3	*OLFML3*	0.92	0.40	0.48
NM_022460.3	HCLS1 binding protein 3	*HS1BP3*	0.44	0.40	0.40
XM_011517212.1	syndecan 2	*SDC2*	0.65	0.41	0.52
NM_001079807.3	pepsinogen 3, group I (pepsinogen A)	*PGA3*	0.38	0.41	0.34
NM_178348.2	late cornified envelope 1A	*LCE1A*	0.39	0.41	0.37
NR_073084.1	cysteine rich protein 2	*CRIP2*	0.52	0.41	0.40
XM_011516973.1	G protein-coupled receptor 20	*GPR20*	0.44	0.41	0.41
NM_001318907.1	tumor protein D52-like 1	*TPD52L1*	1.25	0.41	0.64
XM_011536358.1	fibulin 5	*FBLN5*	0.93	0.42	0.44
NM_001909.4	cathepsin D	*CTSD*	0.88	0.42	0.42

Accession number	Gene title	Symbol	Ratio		
			CAF8	CAF9	CAF15
NM_000823.3	growth hormone releasing hormone receptor	*GHRHR*	0.49	0.42	0.49
NM_004714.2	dual specificity tyrosine phosphorylation regulated kinase 1B	*DYRK1B*	0.46	0.42	0.45
NM_001243598.2	FXYD6-FXYD2 readthrough	*FXYD6-FXYD2*	0.45	0.42	0.42
NM_015982.3	Y-box binding protein 2	*YBX2*	0.63	0.42	0.40
NG_028942.1	phosphatidylinositol binding clathrin assembly protein	*PICALM*	0.44	0.42	0.38
NM_001282529.1	ubiquitin associated protein 2	*UBAP2*	0.44	0.42	0.40
XM_011524792.1	BTB domain containing 17	*BTBD17*	0.71	0.42	0.52
XM_011524133.1	uncharacterized LOC105369205	*LOC105369205*	0.57	0.42	0.44
NM_080645.2	collagen type XII alpha 1	*COL12A1*	0.93	0.42	0.48
NM_003881.2	WNT1 inducible signaling pathway protein 2	*WISP2*	1.22	0.43	0.53
NR_036493.1	peroxisomal biogenesis factor 19	*PEX19*	0.52	0.43	0.47
NR_075070.1	stathmin 3	*STMN3*	0.47	0.43	0.39
XM_006721521.1	arrestin,beta 2	*ARRB2*	0.48	0.43	0.42
NM_005443.4	3'-phosphoadenosine 5'-phosphosulfate synthase 1	*PAPSS1*	0.72	0.43	0.68
NR_134980.1	collagen type XI alpha 1	*COL11A1*	0.78	0.43	0.40
XM_006718518.2	potassium channel tetramerization domain containing 21	*KCTD21*	0.48	0.44	0.42
NM_153340.4	ataxin 7 like 2	*ATXN7L2*	0.49	0.44	0.44
NM_003711.3	phospholipid phosphatase 1	*PLPP1*	0.92	0.44	0.47
XR_927463.1	interferon related developmental regulator 1	*IFRD1*	0.65	0.44	0.55
NM_003407.3	ZFP36 ring finger protein	*ZFP36*	0.50	0.44	0.40
XM_011524812.1	CUE domain containing 1	*CUEDC1*	0.64	0.44	0.49
NM_001318470.1	TSC22 domain family member 3	*TSC22D3*	0.83	0.44	0.47
NM_173482.2	coiled-coil domain containing 105	*CCDC105*	0.45	0.44	0.43
NM_001308279.1	solute carrier family 14 member 1 (Kidd blood group)	*SLC14A1*	1.05	0.44	0.73
NM_002615.5	serpin peptidase inhibitor, clade F (alpha-2 antiplasmin, pigment epithelium derived factor), member 1	*SERPINF1*	0.95	0.44	0.56
XM_011519305.1	nudix hydrolase 5	*NUDT5*	0.88	0.45	0.85
NM_002061.3	glutamate-cysteine ligase modifier subunit	*GCLM*	0.65	0.45	0.62
XM_005273903.3	leucine rich repeat containing 32	*LRRC32*	0.69	0.45	0.50
XM_011524782.1	ADP ribosylation factor like GTPase 4D	*ARL4D*	0.49	0.45	0.44
XM_011520116.1	midkine (neurite growth-promoting factor 2)	*MDK*	0.60	0.45	0.49
NM_002133.2	heme oxygenase 1	*HMOX1*	0.95	0.45	0.48

Accession number	Gene title	Symbol	Ratio		
			CAF8	CAF9	CAF15
XM_005254761.3	Bcl2 modifying factor	*BMF*	0.60	0.46	0.49
NM_005909.3	microtubule associated protein 1B	*MAP1B*	0.61	0.46	0.57
NM_001164098.1	versican	*VCAN*	0.99	0.46	0.77
XM_011514355.1	chromosome 6 open reading frame 223	*C6orf223*	0.48	0.46	0.55
NM_006342.2	transforming acidic coiled-coil containing protein 3	*TACC3*	0.44	0.46	0.51
NM_175723.1	synovial sarcoma, X breakpoint 5	*SSX5*	0.63	0.46	0.49
NM_001308279.1	solute carrier family 14 member 1 (Kidd blood group)	*SLC14A1*	1.09	0.46	0.71
NM_022107.2	G-protein signaling modulator 3	*GPSM3*	0.58	0.46	0.52
NM_001142776.1	ChaC glutathione-specific gamma-glutamylcyclotransferase 1	*CHAC1*	0.49	0.46	0.51
XM_011538373.1	myosin IA	*MYO1A*	0.57	0.47	0.51
NM_001145145.1	solute carrier family 1 member 5	*SLC1A5*	0.70	0.47	0.53
XM_011515689.1	POM121 transmembrane nucleoporin C	*POM121C*	0.51	0.47	0.58
NM_178352.2	late cornified envelope 1D	*LCE1D*	0.48	0.47	0.47
XM_011545784.1	potassium channel tetramerization domain containing 13	*KCTD13*	0.49	0.47	0.45
NM_001195733.1	phosphatidylinositol-4-phosphate 5-kinase, type I, gamma	*PIP5K1C*	0.55	0.47	0.50
XM_005252469.2	phytanoyl-CoA 2-hydroxylase	*PHYH*	0.87	0.47	0.66
NM_001318875.1	flotillin 1	*FLOT1*	0.57	0.48	0.49
XM_005256729.3	phosphoribosyl pyrophosphate synthetase-associated protein 2	*PRPSAP2*	0.53	0.48	0.52
XM_011538436.1	cyclin-dependent kinase 17	*CDK17*	0.53	0.48	0.55
XM_011525661.1	inositol(myo)-1(or 4)-monophosphatase 2	*IMPA2*	0.58	0.48	0.56
XM_006711141.2	CUGBP, Elav-like family member 3	*CELF3*	0.58	0.48	0.51
NM_003247.3	thrombospondin 2	*THBS2*	0.95	0.49	0.49
XR_926244.1	tyrosyl-DNA phosphodiesterase 2	*TDP2*	0.62	0.49	0.51
NR_027265.1	golgi glycoprotein 1	*GLG1*	0.59	0.49	0.81
NM_001042351.2	glucose-6-phosphate dehydrogenase	*G6PD*	0.56	0.49	0.51
NM_021978.3	suppression of tumorigenicity 14	*ST14*	0.48	0.49	0.39
NM_004760.2	serine/threonine kinase 17a	*STK17A*	0.56	0.49	0.60
NM_001004465.1	olfactory receptor family 10 subfamily H member 4	*OR10H4*	0.52	0.49	0.64
NM_001631.4	alkaline phosphatase, intestinal	*ALPI*	0.61	0.49	0.56
NM_001335.3	cathepsin W	*CTSW*	0.64	0.49	0.53
XR_943401.1	estrogen related receptor beta	*ESRRB*	0.54	0.49	0.44
XM_011537796.1	nudix hydrolase 4	*NUDT4*	0.87	0.49	0.55

Accession number	Gene title	Symbol	Ratio		
			CAF8	CAF9	CAF15
NM_018174.5	microtubule associated protein 1S	*MAP1S*	0.54	0.49	0.55
NM_001256716.1	dynein (axonemal) assembly factor 3	*DNAAF3*	0.50	0.49	0.48
